# Genome-wide analysis of Pax8 binding provides new insights into thyroid functions

**DOI:** 10.1186/1471-2164-13-147

**Published:** 2012-04-24

**Authors:** Sergio Ruiz-Llorente, Enrique Carrillo Santa de Pau, Ana Sastre-Perona, Cristina Montero-Conde, Gonzalo Gómez-López, James A Fagin, Alfonso Valencia, David G Pisano, Pilar Santisteban

**Affiliations:** 1 Instituto de Investigaciones Biomédicas “Alberto Sols”, Consejo Superior de Investigaciones Científicas (CSIC) y Universidad Autónoma de Madrid (UAM), C/Arturo Duperier 4, Madrid, 28029, Spain; 2 Memorial Sloan-Kettering Cancer Center, New York, NY, 10065, USA; 3 Centro Nacional de Investigaciones Oncológicas (CNIO), Madrid, Spain; 4 Department of Molecular Biology, Faculty of Science, Nijmegen Centre of Molecular Life Sciences, Radboud University, Nijmegen, HB, 6500, The Netherlands

**Keywords:** Pax8, ChIP-Seq, Expression arrays, CpG island, CTCF, SP1

## Abstract

**Background:**

The transcription factor Pax8 is essential for the differentiation of thyroid cells. However, there are few data on genes transcriptionally regulated by Pax8 other than thyroid-related genes. To better understand the role of Pax8 in the biology of thyroid cells, we obtained transcriptional profiles of Pax8-silenced PCCl3 thyroid cells using whole genome expression arrays and integrated these signals with global cis-regulatory sequencing studies performed by ChIP-Seq analysis

**Results:**

Exhaustive analysis of Pax8 immunoprecipitated peaks demonstrated preferential binding to intragenic regions and CpG-enriched islands, which suggests a role of Pax8 in transcriptional regulation of orphan CpG regions. In addition, ChIP-Seq allowed us to identify Pax8 partners, including proteins involved in tertiary DNA structure (CTCF) and chromatin remodeling (Sp1), and these direct transcriptional interactions were confirmed *in vivo*. Moreover, both factors modulate Pax8-dependent transcriptional activation of the sodium iodide symporter (*Nis*) gene promoter. We ultimately combined putative and novel Pax8 binding sites with actual target gene expression regulation to define Pax8-dependent genes. Functional classification suggests that Pax8-regulated genes may be directly involved in important processes of thyroid cell function such as cell proliferation and differentiation, apoptosis, cell polarity, motion and adhesion, and a plethora of DNA/protein-related processes.

**Conclusion:**

Our study provides novel insights into the role of Pax8 in thyroid biology, exerted through transcriptional regulation of important genes involved in critical thyrocyte processes. In addition, we found new transcriptional partners of Pax8, which functionally cooperate with Pax8 in the regulation of thyroid gene transcription. Besides, our data demonstrate preferential location of Pax8 in non-promoter CpG regions. These data point to an orphan CpG island-mediated mechanism that represents a novel role of Pax8 in the transcriptional output of the thyrocyte.

## Background

Gene regulation has been the subject of intense investigation over the past decades, mainly focusing on detailed characterization of a particular gene or gene family. However, genome-wide mapping of protein-DNA interactions and epigenetic marks is essential for a full understanding of transcriptional regulation. A precise map of binding sites for transcription factors (TFs), core transcriptional machinery, and other DNA-binding proteins is necessary to decode the gene regulatory networks and their contribution to developmental processes and human disease [1]. In fact, regulation of gene expression by TFs is one of the major mechanisms for controlling cell proliferation, differentiation, and function.

To elucidate the mechanism(s) operating in the establishment and maintenance of cell-specific differentiation, we used thyroid epithelial cells as a model system. These cells are the largest cell population of the thyroid gland and express different TFs called Nkx2.1, Foxe1, Hhex and Pax8, which define the thyroid differentiated phenotype [2,3]. It is well known that these factors bind to the promoter regions of thyroid-specific genes, such as the genes encoding Thyroglobulin (*Tg)*, Thyroperoxidase (*Tpo*), and the Sodium Iodide Symporter (*Nis*), thus regulating their expression. Nevertheless, despite the key relevance of these TFs for thyroid biology, few studies have described additional *loci* that are transcriptionally regulated by the above mentioned TFs, nor have sequences been described to which these factors bind in enhancers, silencers, or boundary elements that could potentially regulate the transcription of genes over large distances.

Among these thyroid TFs, Pax8 is a member of the paired box-containing proteins and is expressed in the thyroid and kidney, and in the central nervous system during development [4]. It plays an essential role in the differentiation of thyroid cells and, according to the phenotype of *Pax8* knockout mice, it seems to be responsible for the formation of the follicles of polarized epithelial thyroid cells [5]. Also, the association between mutations of *PAX8* and congenital hypothyroidism in humans underlines an important function of this transcription factor in thyroid pathologies [6]. In order to better understand its role in the maintenance of thyroid function, we explored the transcriptional profile of Pax8-silenced thyroid cells, and integrated these signals with global cis-regulatory sequencing studies (chromatin immunoprecipitation followed by sequencing; ChIP-Seq).

The ChIP-Seq strategy allowed us to identify a large number of novel *in vivo* Pax8 binding sites that were significantly associated with CpG islands or high GC content sequences. Interestingly, immunoprecipitated peaks were mainly located along intronic regions and grouped in distal positions with respect to transcriptional start sites. Consensus sequence screening of these areas suggested Pax8 interaction with several core transcriptional elements (motif ten element, Inr, and BRE), transcription factors belonging to the AP1 family, and trans-elements factors involved in high order chromatin structure (CTCF) and remodeling (Sp1). Co-immunoprecipitation and reporter assays demonstrated both physical binding and transcriptional cooperation between CTCF/Sp1 and Pax8. Combining sequencing and expression array data, we ultimately provided insights into Pax8*-*transcriptional networks in the differentiated thyroid that predict its involvement in relevant biological processes and pathways.

## Results

### Genomic features associated with Pax8 binding sites

In order to identify the genome-wide binding patterns of Pax8 in differentiated thyroid cells, we performed ChIP-Seq in PCCl3 rat thyrocytes using IP and non-IP conditions. Prior to massive sequencing, both conditions were interrogated to verify Pax8 binding site enrichment by means of semi-quantitative PCR (Additional file 1). Using this approach, we confirmed DNA immunoprecipitation of Pax8 binding regions in the rat *Nis* and *Tpo* promoters (Additional file 1A), as previously described [7-9]. Therefore, we considered both IP and non-IP conditions as useful samples to further identify whole genome Pax8 binding sites by means of high throughput sequencing technology. After sequencing analysis, we obtained 11,613,355 and 12,125,758 raw reads for control and IP conditions, respectively. Of these, 6,714,002 (57.8%) and 6,431,519 (53.0%) fulfilled the ≤2 mismatches quality filter.

To further localize regions of Pax8 enrichment, we identified Pax8 peaks genome-wide. Peak detection analysis using MACS defined 13,151 Pax8-enriched regions with an average length of 681 bp (Additional file 2). Visual inspection of the Pax8 binding sites and the profiling data in a genome browser for well-known Pax8 targets like *Nis, Tpo, *[7,9] and *WT1* (Wilms' tumour gene 1) [10], showed Pax8 binding sites close to the 5'-UTRs of these genes as previously described. A detailed analysis of *Nis* (*Slc5a5*, Na^+^/I^−^ symporter), whose transcription status is tightly regulated by Pax8 [7], showed a significant Pax8 binding site overlapping with the *Nis* upstream enhancer (Additional file 1B). These findings clearly validated ChIP-Seq as an efficient and powerful technique for mapping Pax8 binding sites in PCCl3 cells.

**Figure 1:**
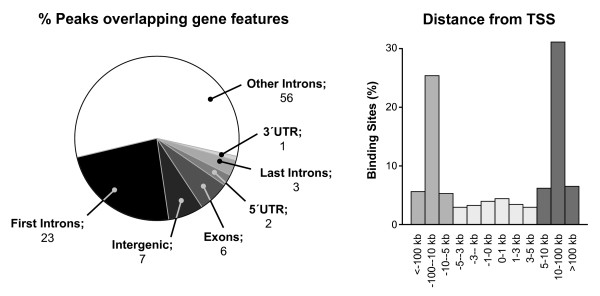
**MACS IP peaks characteristics.** Diagrams show percentage of peaks overlapping with gene features (left), or their location with regard to the closest transcription start site (TSS) (right).

**Figure 2:**
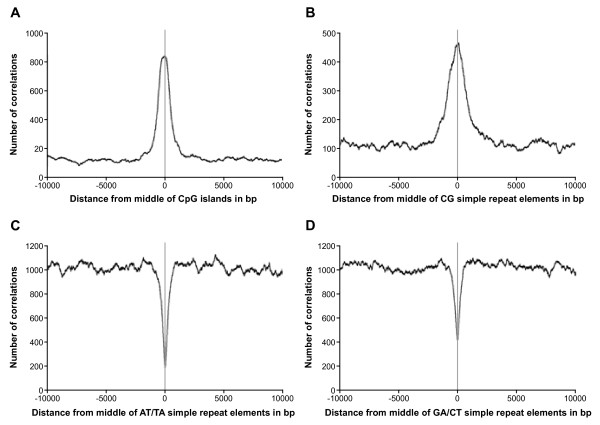
**Pax8 immunoprecipitation enrichment.** Graphs displaying number of correlations versus the distance from middle of CpG islands (panel **A**), CG (panel **B**), AT/TA (Panel **C**) and GA/CT (panel **D**) simple repeat elements as determined by the MACS ChIP-Seq program.

Association of Pax8 enriched regions with annotated genomic features indicated that Pax8 tends to localize within intronic regions (82%); only a few peaks revealed binding to coding (6%) or 5'-UTR regulatory regions (2%) (Figure 1). In addition, Pax8 binding sites showed preferential binding to regions located 10-100 kb upstream or downstream of the closest transcription start site (Figure 1). We also assessed the general sequence content of these peaks, including CG content and dinucleotide frequencies. We found a clearly increased correlation between Pax8 binding sites and CpG islands (Figure 2A) and CG simple repeat elements (Figure 2B) in comparison with other dinucleotide combinations throughout the rat genome (Figure 2C and 2D). All these data suggest preferential Pax8 interaction with “orphan” CGIs, CG-rich intragenic elements not associated to 5'-UTR regions [11].

### Pax8 immunoprecipitated regions delineate Pax8 consensus core sequence *in vivo*

**Figure 3:**
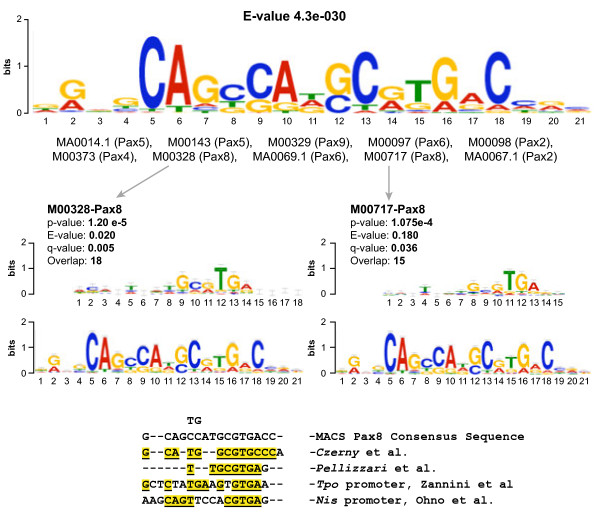
**ChIP-Seq peaks delineate*in vivo*Pax8 binding site.** Image depicts most significant motif defined by the MEME program using the 500 most relevant MACS ChIP-Seq immunoprecipitated peaks and its comparison with Pax proteins-DNA binding consensus sites defined using in vitro approaches (Czerny et al; Pellizzari et al) [13,14] and previously known Pax8 targets, Nis and Tpo respectively (Ohno et al; Zannini et al) [7,9]. Underlined characters highlighted in yellow are nucleotides similar between our Pax8 motif and the other consensus sequences.

We considered the most significant Pax8 peaks (Additional file 3) to evaluate how efficiently ChIP immunoprecipitated the Pax8-DNA binding sequence. Among the most significant consensus motifs obtained using the MEME-chip and TOMTOM *in silico* tools, we observed a significant overrepresentation of Pax-related binding sites, including sites for Pax8 and members of its own subfamily (Pax2 and Pax5) (Figure 3). The Pax8 binding motif here defined encompasses motifs obtained by individual-gene based approaches, such as those defined for rat and human *TPO*[9,12] and for the rat *Nis* upstream enhancer [7] (Figure 3), as well as those defined by *in vitro* studies outlining the binding sequence for Pax8 [13] and the Pax2/5/8 subfamily (AAGCGTGAC) [14]. Of note, our study is the first to describe the *in vivo* Pax8 binding sequence derived from its DNA binding along the whole rat genome.

### Immunoprecipitation data reveals interaction of Pax8 with various TFs

To identify possible interactions between Pax8 and other TFs, Genomatix suite screening was performed to identify the most over-represented motifs in the Pax8 binding regions identified by MACS [15]. Overrepresented consensus motifs in Pax8 peaks, as well as fold change and z-score values are shown in Table 1 and Additional file 4. These analyses showed an overrepresentation of the Pax5 (V$Pax5.1 z-score = 87.59, overrepresentation: 2.86; V$Pax5.2 z-score = 78.62, overrepresentation: 3.95) and Pax9 consensus motifs (V$Pax9.1 z-score = 73.71; overrepresentation: 3.63) with a modest association for Pax8 (V$Pax8.1 z-score = 5.46, overrepresentation: 1.07). These results may be explained by the DNA binding similarities among members of the same Pax subfamily (Pax2, Pax5, and Pax8).

**Table 1:** Main DNA binding motifs overrepresented in Pax8-dependent peaks

**TF Matrices**	**Family information**	**Matches in Input (n)**	**Expected (genome)**	**Overrep. (genome)**	**Z-Score (genome)**
O$BRE.01	Transcription factor II B (TFIIB) recognition element	924	88.75	10.41	88.61
O$HMTE.01	Human motif ten element	2481	350.02	7.09	113.88
O$XCPE1.01	X gene core promoter element 1	5454	1531.85	3.56	100.21
O$DMTE.01	Drosophila motif ten element	4415	1442.3	3.06	78.27
O$INR_DPE.01	Initiator (INR) and downstream promoter element (DPE) with strictly maintained spacing	2460	1071.16	2.30	42.42
V$NRF1.01	Nuclear respiratory factor 1	6640	1323.52	5.02	146.13
V$SP1.02	GC-Box factors SP1/GC; Stimulating protein 1, ubiquitous zinc finger transcription factor	5792	1090.23	5.31	142.39
V$SP1.03	GC-Box factors SP1/GC; Stimulating protein 1, ubiquitous zinc finger transcription factor	4802	1129.17	5.14	139.05
V$ZF5.01	Zinc finger / POZ domain transcription factor	5543	1260.88	4.40	120.59
V$CTCF.01	CCCTC-binding factor	4729	1015.47	4.66	116.52

The *Overrep. (genome)* column represents the fold overrepresentation of these matrices in our IP peaks compared to their presence in the rat genome, and the *Z-Score (genome)* column indicates association value between the DNA matrices and Pax8-immunoprecipitated DNA.

**Figure 4:**
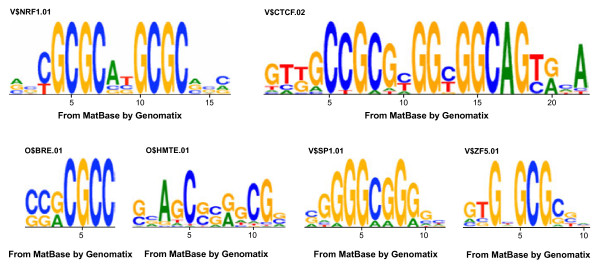
**Core matrices obtained for different significantly overrepresented transcription factor DNA-binding sequences in Pax8 immunoprecipitated peaks.** Matrices shown include both transcription factors (V$NRF-1.01, V$CTCF.02, V$SP1.01 and V$ZF5.01) and transcriptional core elements (O$HMTE.01 and O$BRE.01).

In agreement with previous reports describing interactions between members of the Pax family and AP1 factors [16], we also observed enrichment of transcription factors belonging to this latter family (V$NRF-1, nuclear respiratory factor-1; z-score = 146.13, overrepresentation = 5.02). Intriguingly, several transcription factor matrices (V$CTCF, V$ZF5 and V$SP1F) and general transcriptional regulatory elements (O$BRE, O$INR-DPE, and *Drosophila melanogaster* and *Homo sapiens* Motif ten elements (O$DMTE and O$HMTE)) showed significant association with Pax8 IP peaks (Table 1). In order to rule out any nonspecific effect of the Pax8 antibody on the recognition of these transcription factors not related to Pax8, we compared their amino acid sequences with the Pax8 protein by means of the DNAStar alignment program. No significant similarities were observed among the considered proteins, thus ruling out any unspecific binding of the Pax8 antibody (data not shown). As shown in their corresponding IP Genomatix motifs (Figure 4), all these transcription factors preferentially bind to regions with high GC content, which could be related to the association of Pax8 to CpG islands and CG repeats.

**Figure 5:**
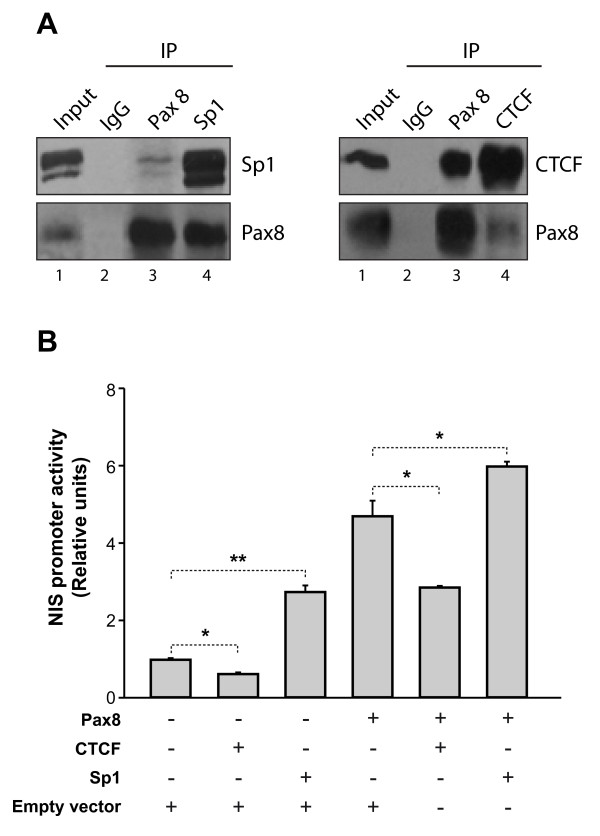
**CoIP assays demonstrating physical interaction of Pax8 with Sp1 and CTCF. A)** Nuclear extracts from control or hCTCF-transfected PCCl3 cells were obtained and immunoprecipitated (IP) with anti Pax8, anti-Sp1 or anti-CTCF antibodies. Immunoblotting was performed against Sp1 (top, left panel), CTCF (top, right panel) or Pax8 (bottom, left and right panels). Lanes 1 are the input and lanes 2 are the nonspecific IPs using IgG. The Figure shows a representative Western-Blot. **B)** Reporter assays were performed using the pNIS-2.8 promoter and expression vectors as indicated in the figure. Promoter activity is expressed as fold induction, relative to the activity observed in the presence of empty expression vector. The amount of total DNA used for each transfection was adjusted with the matched empty vector control to 1 μg. Luciferase activity was normalized to renilla activity derived from the cotransfected pRL-CMV vector to adjust for transfection efficiency. Results are mean ± SD of three independent experiments. * *p*-value < 0.005; **, *p*-value < 0.01.

To validate the interactions between Pax8 and the TFs described before, we performed co-immuprecipitation assays in PCCl3 cells using specific antibodies for Pax8, Sp1 and CTCF. As shown in Figure 5A, both CTCF and Sp1 coprecipitate with Pax8, confirming physical binding among these transcription factors *in vivo* and suggesting the existence of common transcriptionally regulated targets. Given that the *Nis* promoter region conferring regulation by Pax8 (NIS upstream enhancer) overlapped with potential sites for both transcription regulators (data not shown), we performed transfection experiments in HeLa cells using a reporter construct containing the *Nis* promoter [17]. As shown in Figure 5B, Sp1 strikingly increased NIS transcriptional activity, while cotransfection of Pax8 and Sp1 resulted in a synergestic effect on promoter activation. On the other hand, contransfection with CTCF induced a statistically significant decrease in transcription (Figure 5B). These data unequivocally demonstrate a functional cooperation between Pax8 and Sp1 and CTCF in transcriptional regulation.

### Expression arrays analysis identifies a wide set of *loci* regulated by Pax8

We used whole genome expression arrays to identify Pax8-regulated genes by comparing expression profiles of Pax8-silenced PCCl3 cells with both scrambled siRNA-treated and wild type (wt) PCCl3 cells. This last condition was included to consistently integrate both expression array signals and global cis-regulatory sequencing studies into the same experimental conditions. Misinterpretation of expression data due to compensatory effects via Pax8-related paralogues (Pax2 and Pax5) is ruled out, given that both transcription factors are not expressed in thyroid cells.

Regarding the comparison of si*Pax8*-PCCl3 vs. wt PCCl3, 3,035 and 3,354 probes were down and up-regulated in the Pax8-silenced condition, respectively (Additional file 5). A lower number of significant probes was detected for si*Pax8*-PCCl3 vs. siScramble-PCCl3 (797 and 777 probes were down and up-regulated in the Pax8-silenced condition, respectively) (Additional file 5). Statistically significantly differently expressed probes (adjusted *p*-values <0.005) for both comparisons included 633 down and 565 up-regulated targets (Additional file 5), which represent a set of 849 *loci*.

### Pax8 is involved in controlling key cellular events

Gene lists were ranked based on t-statistics for gene set enrichment analysis. The most significant GO terms and adjusted *p*-values for both array comparisons are shown in Table 2, and more detailed data, including genes belonging to each significant category, are listed in Additional files 6, 7 and 8. In general, the FatiScan tool revealed significant association for biological processes related to immune response, molecule transport, response to stimuli, cell motion/adhesion, cell proliferation, and translational processes. In relation to this last term, ribosome-related GO classes were also observed for other FatiScan categories: molecular processes (structural constituent of ribosome; adjusted *p*-value: 4.43e-13), KEGG pathway (rno-03010; adjusted *p*-value: 8.7e-6), and cell component analysis (downregulation of ribosomes and ribonucleoprotein-related genes; GO: 0005840 and 0030529; adjusted *p*-value: 1.9e-5) (data not shown).

**Table 2:** FatiScan gene set enrichment analysis

**Gene Ontology term**	**WT vs Pax8** **adj*p*-value**	**Scramble vs Pax8** **adj*p*-value**
Translation (GO:0006412)	7.10E-12	1.91E-09
Response to external stimulus (GO:0009605)	2.20E-05	2.02E-07
Response to wounding (GO:0009611)	2.20E-05	6.87E-05
Cellular component movement (GO:0006928)	4.97E-03	6.87E-05
Response to hormone stimulus (GO:0009725)	2.86E-02	1.24E-04
Immune response (GO:0006955)	9.08E-11	1.42E-04
Cell adhesion (GO:0007155)	8.00E-03	6.95E-04
Response to steroid hormone stimulus (GO:0048545)	2.39E-03	1.99E-03
Antigen processing and presentation (GO:0019882)	2.00E-05	3.13E-03
Cell migration (GO:0016477)	3.16E-03	3.65E-03

Most significant Gene Ontology (GO) terms overrepresented in both expression array comparisons. adj *p*-value: adjusted *p*-value.

### Single functional analysis

We additionally used the FatiGO *in silico* tool to extract Gene Ontology (GO) terms overrepresented in our down- and up-regulated set of differentially expressed genes. Considering down-regulated probes for each comparison, we observed an enrichment in biological processes related to a wide variety of DNA, RNA, and protein processes (purine and pyrimidine metabolism, response to DNA damage, DNA replication, nucleotide and base exchange repair, mismatch repair and homologous recombination, RNA degradation, and amino acid metabolism), cell response to chemical and stress stimuli, immune response, and p53 and insulin-related pathways (phosphatidyl inositol system and metabolism) (Additional file 9). Concerning GO terms enriched amongst up-regulated probes, it is worth to mention the over-representation of genes involved in biological processes such as immune response, cell response to stimuli, apoptosis and cell death, cell motion/migration/adhesion, and regulation of cell differentiation (Additional file 10).

Table 3 and Additional file 11 depict the most significant and complete set of KEGG pathways overrepresented in under and overexpressed target genes, respectively. KEGG pathways significantly enriched in these Pax8-regulated genes included vesicle-related terms (endocytosis, rno004144; lysosomes, rno004142), DNA/RNA events, cell cycle, cell-cell interactions (focal adhesions, adherens junctions), cancer-related pathways (MAPK, JAK-STAT, p53, ERBB, TGFβ, and VEGF), amino acid metabolism, and insulin/inositol phosphate signalling events.

**Table 3:** KEGG pathways associated to Pax8 silencing

Global class		KEGG pathway	Scr. vs Pax8 adj*p*-value	Wt. vs Pax8 adj*p*-value
Downreg. probes	Phosph. I.	Phosphatidylinositol signaling system	1.28E-03	8.10E-05
		Inositol phosphate metab.	4.18E-02	9.88E-04
	Aa metab.	Glycine, serine and threonine metab.	1.25E-04	2.42E-02
		Selenoamino acid metab.	1.61E-03	1.16E-02
		Cysteine and methionine metab.	7.21E-03	1.25E-03
		Arginine and proline metab.	1.06E-02	9.73E-07
	Cell cycle	Cell cycle	9.67E-05	9.73E-07
	CAMs	Cell adhesion molecules	9.37E-03	5.39E-03
	Immune response	Antigen processing and presentation	1.56E-05	2.86E-03
		Autoimmune thyroid disease	5.26E-04	2.76E-03
	DNA/RNA processes	Purine metab.	1.56E-05	9.52E-09
		Base excision repair	5.85E-05	2.20E-03
		Pyrimidine metab.	1.25E-04	5.86E-09
		Nucleotide excision repair	2.99E-04	3.22E-03
		RNA degradation	9.95E-04	2.76E-03
		Mismatch repair	1.22E-03	4.76E-05
		Homologous recombination	2.30E-02	7.50E-03
		DNA replication	2.88E-02	2.98E-06
	Signaling pathways	p53 signaling pathway	7.24E-03	7.26E-07
Upreg. probes	Cell processes	Endocytosis	1.13E-10	4.66E-14
		Lysosome	1.23E-06	1.55E-05
	Cell migration/adhesion	Cell adhesion molecules	2.43E-09	1.59E-06
	Signaling pathways	Cytosolic DNA- sensing pathway	4.36E-04	5.70E-06
		NOD-like receptor signaling pathway	2.59E-03	9.17E-05
		Toll-like receptor signaling pathway	4.26E-06	3.52E-07
		Chemokine signaling pathway	1.91E-05	9.22E-06
		MAPK signaling	7.52E-06	4.69E-07
	Cancer	Thyroid cancer	1.65E-02	1.23E-04
		Prostate cancer	2.30E-05	4.74E-07
		Endometrial cancer	8.49E-04	1.49E-05
		Pancreatic cancer	1.34E-05	7.79E-06
		Renal cell carcinoma	1.95E-03	9.22E-06
		Colorectal cancer	1.16E-03	4.71E-05
		Pathways in cancer	5.20E-06	2.07E-10
	Immune response	Viral myocarditis	2.49E-11	2.68E-08
		Graft-versus-host disease	1.53E-09	5.16E-07
		Allograft rejection	9.99E-08	4.28E-06
		Autoimmune thyroid disease	2.53E-08	1.59E-06
		Antigen processing and presentation	1.13E-10	5.06E-13

Most significant KEGG pathways enriched among common downregulated (n = 633) and upregulated (n = 565) probes for both expression array comparisons. Downreg.: Downregulated; Upreg.: Upregulated; adj *p*-value: adjusted *p*-value; Phosph.I:Phosphatidylinositol; Aa: Amino acid; metab.:metabolism.

### Integrated data reveal a reduced percentage of genes transcriptionally regulated through promoter sequences

**Figure 6:**
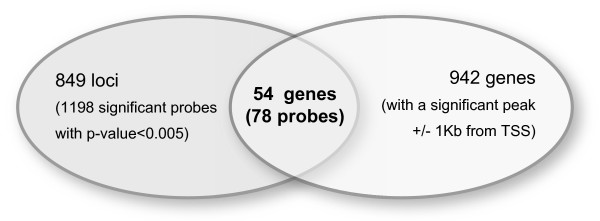
**Venn diagram showing the number of significant*loci*obtained for each screening (expression arrays and ChIP-Seq) and for combined analysis.** 78 significant probes (29 and 49 up and down-regulated, respectively, in the si*Pax8* PCCl3 condition) showed a *p*-value <0.05 in the expression arrays and at least one immunoprecipitated peak within 1 kb of a TSS.

To answer the question whether independent Pax8 binding to the genomic regions has functional consequences through changes in the expression level of target genes, the ChIP-Seq data were integrated with the gene expression profiling data. As shown in the Venn diagram (Figure 6), 78 differentially expressed probes (29 and 49 up and down-regulated, respectively, in the si*Pax8* PCCl3 condition) representing 54 *loci* were associated with genes showing a significant peak around +/− 1 kb from a TSS. This number of genes represents 6.4% (54 out of 849 genes) of the significantly associated genes identified by expression arrays (Additional file 5). This small overlap can be explained by indirect effects of Pax8 or by the binding of Pax8 to “orphan” CGI regulatory elements, also sites of transcriptional initiation but not related with TSSs or promoter regions [11]. It is worth mentioning that we identified neighbouring genes showing significantly altered expression according to our expression data, which could be simultaneously regulated by a unique Pax8 binding site; for example, the closely positioned genes *Padi1* and *Padi3* (peptidyl arginine deiminase, types I and III) on chromosome 5, and *Mlph* (melanophilin) and *Rab17* on chromosome 9 (Additional file 12).

### Independent validation confirms significant findings defined by ChIP-seq and expression arrays

**Figure 7:**
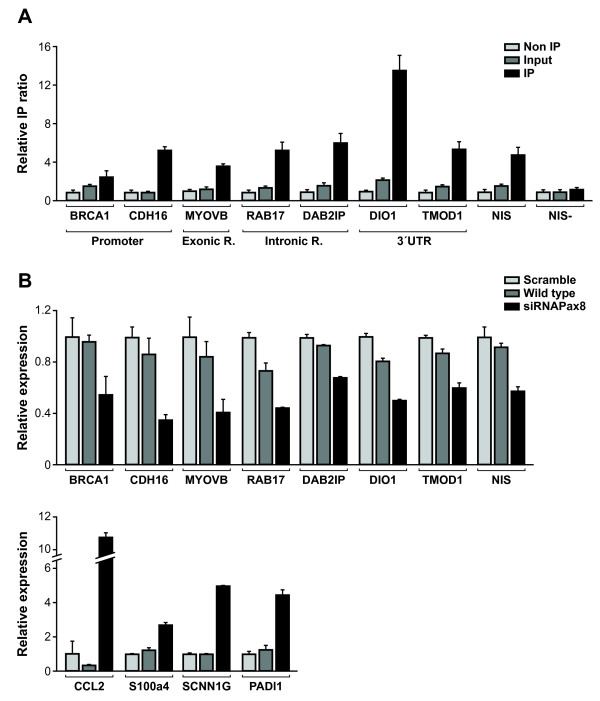
**Experimental validation of ChIP-Seq. A)** Normalized IP ratio (arbitrary units) of corresponding DNA sequences belonging to significant peaks of 7 representative genes found in the present analysis. The NUE element of rat *Nis* (NIS) was used as a positive control while an unspecific sequence of the same *Nis* promoter (NIS-) was used as a negative control. **B)** Relative expression assessed by means of qRT-PCR of 7 (upper pannel) and 4 (lower pannel) genes differentially down- and upregulated in Pax8-silenced PCCl3 cells (siRNA Pax8) *vs*. wild type (wt)/siScramble (Scramble) cells, respectively. As a Pax8-dependent positive control, we evaluated *Nis* mRNA expression levels.

We performed experimental validations using independent immunoprecipitated and siRNA silenced PCCl3 samples, as well as their corresponding controls. Among the genes included for this validation were: *Brca1* (breast cancer 1), a well-known tumour suppressor gene involved in the maintenance of genomic stability and related to breast carcinoma development [18]; *Cdh16* (cadherin 16, KSP-cadherin), a member of the cadherin protein family acting as a morphogenic factor for tissue development [19] and recently described to be regulated by Pax8 [20,21]; *Rab17* (a member of the RAS oncogene family) and *Myo5b* (myosin VB), genes previously described to be involved in epithelial vesicle trafficking in highly polarized cells [22,23]; *Dab2ip* (DAB2 interacting protein), a tumour and metastasis suppressor gene which encodes a Ras GTPase-activating protein [24]; *Dio1* (iodothyronine deiodinase, type I), an essential gene for thyroid hormone action given that it codes for an oxidoreductase involved in thyroid hormone activation by converting the prohormone T4 into bioactive 3,5,3'-triiodothyronine (T3) [25] and *Tmod1* (tropomodulin 1), a gene encoding a protein which inhibits actin filament elongation and that is consequently involved in cytoskeleton structure regulation and cell morphology [26]. As shown in Figure 7A, after Pax8 chromatin immunoprecipitation followed by semiquantitative RT-PCR, we observed an enrichment of IP regions for all the above-mentioned validation targets in comparison with both non-IP samples and input samples.

In addition, Pax8 silencing by means of transient transfection of siRNA was significantly associated with decreased expression levels of these potential targets (Figure 7B, upper pannel), thus demonstrating a direct transcriptional effect of Pax8 on these genes. mRNA expression validation was also done for several genes that were upregulated in the absence of Pax8, including the genes encoding: CCL2, a chemokine involved in thyroid autoimmunity [27]; S100A4, a calcium-binding protein which plays a role in angiogenesis, extracellular matrix remodelling and tumor microenvironment, and reported to be overexpressed in metastatic papillary thyroid microcarcinomas [28]; SCNN1G and PADI1, which exert a role in Na^+^ transport and differentiation in epithelial cells, respectively [29,30] (Figure 7B, lower panel). *In silico* analysis of significant IP peaks located along promoter areas of these *loci* demonstrated Pax8 potential binding sites in 3 out of 4 genes (data not shown). Globally, these findings underscore the efficiency and accuracy of ChIP-Seq and expression array technologies to define a Pax8-dependent gene network, which allowed us to identify biological functions of Pax8 in thyroid cells.

## Discussion

Despite the known relevance of the transcription factor Pax8 for adult thyrocyte physiology, few data have been published concerning Pax8 target genes other than key thyroid-related genes (*TgTpo*, and *Nis*). The transcriptional output of Pax8 during thyroid development is unknown but essential, given that thyroid follicular precursors are not formed in *Pax8* null mouse embryos, which ultimately impairs the formation of follicle structures and thyroid hormone biosynthesis [5].

With regard to its link to tumour development, *Pax8* expression decreases or is lost in follicular thyroid carcinomas as well as in oncogene-transformed thyroid cells [31]. Moreover, several well-known tumour suppressors, including *TP53*[32] and *WT1*[33], have been defined as Pax8 targets, and cytoplasmic Pax8 staining has been positively associated with tumour size, metastasis, local invasion, recurrence, or persistence in the thyroid [34]. Taking into account all these premises, and in order to better understand the role of Pax8 in the maintenance of thyroid function, we decided to explore the transcriptional profile of Pax8-silenced thyroid PCCl3 cells, and to integrate these signals with genome wide cis-regulatory studies. Thus, our experimental design combined putative and novel Pax8 binding sites with analysis of actual target gene expression regulation, a strategy successfully used for identifying direct targets for other transcription factors [35,36].

Our unbiased mapping of Pax8 binding sites along the rat genome has identified a large number of DNA sequences that are occupied in living thyrocytes. Moreover, this is the first study addressing *in vivo* genome-wide mapping of Pax8-DNA binding sites, and the Pax8 consensus binding motif here defined encompasses motifs described by previous reports focused either on single gene regulation [7,12] or on Paired-box DNA motif characterization [13,14]. The ChIPSeq approach also led to significant immunoprecipitation of genomic sequences containing CpG islands, as well as CpG dinucleotides. Extensive literature has linked the location of CpG islands and GC-enriched regions to transcriptionally permissive chromatin [37,38], which could lend support to a relevant role of Pax8 in the transcriptional output of the thyrocyte. About half of all CpG islands self-evidently contain TSSs, while the other half (known as “orphan” CpG islands) are either within or between characterized transcription units and have unknown significance [11,38]. Despite a lack of association to annotated promoters, “orphan” CpG islands have been associated to transcriptional initiation and dynamic expression during development [39]. In agreement with this, we found significant Pax8 binding to orphan CpG islands in intronic regions and a preferential binding to such islands 10–100 kb upstream or downstream of a transcription start site. In fact, genomic studies indicate that almost half of the human coding genes have alternative promoters [40] and that transcription factor binding sites (TFBSs) in classically defined promoter regions may represent a minority of genomic binding sites [41]. Moreover, this latter report clearly demonstrated an association between TFBSs and the expression of non-coding RNAs, which could be modulating the expression of the gene encoded by the opposite strand. Less directly, a subset of intergenic H3K4me3 peaks, many of which are likely to correspond to orphan CpG islands, were found to represent TSSs for long non-coding RNAs [42]. Our findings suggest that Pax8 binds orphan CpG islands that could represent alternative promoters of nearby annotated genes [43] or ncRNAs that regulate gene expression.

Otherwise, Pax8-dependent ChIP-Seq data demonstrated an enrichment of genomic regions with overrepresentation of general transcriptional regulatory elements (Human MTE and Drosophila MTE, Inr-DPE and BRE). MTE constitutes a core promoter element (~20-30 nt downstream of the TSS) associated with RNA polymerase II-mediated transcription [44,45]. Furthermore, human orphan CpG islands have been associated with RNA polymerase II binding sites [39]. On the other hand, Inr-DPE and BRE elements represent functional binding sites for TFIIB and TFIID (transcription initiation factor IIB and IID, respectively), which are main components of the basal transcription machinery [46]. Interestingly, Jin *et al* recently described synergistic MTE-Inr-BRE transcriptional modules in more than 9,000 orthologous mouse and human genes [47]. Whereas functional experiments should be performed to demonstrate an interaction of Pax8 with these general core elements, our data underscore the importance of synergistic interactions between core promoter elements and tissue-specific TFs to ultimately modulate gene expression.

### Potential Pax8 partners in transcriptional regulation

2Apart from the classical view of TFs interacting with promoter regions, TFs could activate gene expression by interacting with common lineage-specific TFs and/or binding to distal regions (enhancers). Synergistic effects of Pax8 and AP1 proteins have been shown to occur in the regulation of *Nis* transcription through interaction along the NUE element [7], and AP1 and PAX proteins also interact to cooperate in the modulation of transcription of other genes [16]. Accordingly, we observed an overrepresentation of binding motifs related to NRF-1 (Nuclear respiratory factor-1), and several AP1 members (c-FOS, BATF3, and c-JUN) were differentially expressed in Pax8-deprived thyroid cells. However, no significant findings were obtained for other transcription factors described to act synergistically with Pax8, such as Nkx2-1 and TAZ/WWTR1 proteins [48], indicating that this cooperative transcriptional role could be restricted to specific *loci* rather than representing a global transcription phenomenon in thyroid cells.

Functional studies described in the present paper confirmed physical *in vivo* interactions between Pax8 and CTCF or Sp1 in thyrocytes. These novel partners were further demonstrated to modulate the effect of Pax8 on the transcription of the *NIS* gene, thus confirming that these interactions are functionally relevant. Evidence has been accumulating concerning the role of CTCF in the establishment of intra-chromosomal loops which ultimately mediate protein-protein contacts between distal complexes and the general transcription machinery [49,50]. On the other hand, Sp1 is a ubiquitously expressed transactivator, which physically interacts with several components of TFIIB and TFIID (mentioned above as potential Pax8 interacting proteins) and factors related to epigenetic events, such as histone deacetylases and p300/CBP histone acetyltransferase [51]. Interestingly, several studies have described synergistic interactions between Pax8 and p300 acetyltransferase for enhancing the transcriptional activity of thyroid-related genes [52,53]. Taking into account this complete transcriptional scenario, our data describe potential interactions of Pax8 with both common TFs and core elements, which could cooperate in chromatin remodeling for transcriptional regulation in thyroid cells.

### Identification of biological processes controlled by Pax8 in thyroid cells

Pax8 has been mainly associated to thyroid differentiation and development through its transcriptional role in key thyroid-related genes [54,55]. At this regard, we observed a downregulation of *DIO1* after abolishing Pax8 (Additional file 5), which potentially binds to a critical region for selenocystein insertion in the *DIO1* mRNA. Data were recently provided indicating that TSH tightly regulates DIO1 expression in thyroid cells through Pax8-dependent *DIO1* mRNA stabilization (S.G. Leoni; unpublished observations). Moreover, gene expression profiling in normal versus malignant thyroid tissues demonstrated a downregulation of DIO1 and DIO2 [56], which could be linked to Pax8 loss during cancer progression.

Intriguingly, Pax8 modulates the expression of several genes involved in carcinogenesis and thyroid malignancies (phosphatidyl-inositol/insulin and MAPK pathways) and cell cycle processes (*CDKN2BCCNB1* and *CCNB2*, among others) (Additional file 11). These findings are in accordance with previous studies in which Pax8 expression was abolished in the differentiated thyroid cell line FRTL5 [20,57]. Our data would also explain the biological mechanism underlying the partial decrease in thyrocyte proliferation in response to both IGF-I and TSH (main regulators of thyroid proliferation and differentiation) after both *Nkx2.1* and *Pax8* mRNA silencing [57].

DNA-related biological processes involved a plethora of functional categories (replication, repair and metabolism), highlighting the novel finding of *Brca1*-dependence on Pax8. In this regard, Shih *et al* described that *BRCA1* and *BRCA2* germline mutations were twice as common in individuals developing a second non-ovarian carcinoma, with follicular thyroid carcinoma being one of the most frequent secondary tumours [18]. This finding can be of great relevance in the development of sporadic thyroid tumors, given that, as mentioned before, *Pax8* expression is decreased or lost in thyroid tumours.

Recent reports have associated the transcription factors Pax2 and Pax5 with increased capabilities for cell motility and adhesion in human cancer [58,59]. In parallel with these Pax-related functions, we observed significant expression changes of *loci* involved in cell motion/adhesion, notably the Pax8 effect on *NCAM1* (neural cell adhesion molecule 1) transcription. NCAM1 and other components of adherens junctions, such as cadherins, have been described to be essential for maintaining cell polarity and epithelial integrity [60]. Interestingly, Cadherin-16 (Cdh16/Ksp-cadherin) was recently proposed to play a TSH-regulated role in thyroid development [61], and its expression and promoter activity is controlled by Pax8 [20,62]. We have not only confirmed transcriptional regulation of *Cdh16* by PAX8, but also defined additional PAX8-dependent genes that could be essential for thyroid cell polarity (*MYO5b* and *Rab17*, among others). In this regard, germline mutations in *MYO5b* have been associated with disruption of epithelial cell polarity in MVID (MIM251850) [23]. This role is exerted via its involvement in vesicle trafficking through direct interactions with Rab GTPase proteins, such as RAB11a and RAB8a. Further functional studies should be performed to evaluate potential Myo5b interactions with RAB17, another Rab GTPase protein involved in membrane trafficking and confirmed as a Pax8 target in the present study.

## Conclusions

State-of-the-art cis-regulatory sequencing studies have been combined with mRNA silencing and expression arrays to further characterize the functional relevance of TF-interacting DNA regions and thus to define their transcriptional output. In our study, we describe Pax8 as a master regulator of key cellular processes for thyrocyte biology, including cell cycle regulation, DNA repair, replication and metabolism, and cell polarity, and define a large set of genes whose expression is modulated by Pax8. However, only a minor fraction (6.4%) of the Pax8 binding sites identified are close to TSSs and correlate with altered mRNA expression, in agreement with studies carried out on other TFSs (1-10%) [35,36]. This moderate percentage may be explained by the Pax8 binding site distribution, where most of the binding sites are related with orphan CGI regions. In this regard, our study demonstrates Pax8 binding sites in regions distal to TSSs, preferentially in intronic regions, which highlights a potential role as a distal or alternative transcriptional regulator, although this does not rule out indirect regulation. Distal regulation by Pax8 is supported by the interaction with chromatin remodeling factors such as CTCF and Sp1 described in the present study. Therefore, these findings suggest a new function of Pax8 as a chromatin remodeling factor in thyroid follicullar cells, which should be validated and elucidated in future studies.

## Methods

### Cell culture and plasmids

PCCl3 cells are a continuous line of thyroid follicular cells derived from Fischer rats that express the thyroid-specific genes *TgTpo*, and *Nis*, as well as the thyroid-specific transcription factors Nkx2.1, Foxe1, and Pax8 [63]. They were grown in Coon’s modified Ham’s F-12 medium supplemented with 5% donor calf serum and a six-hormone mixture [64]. For transfection assays, HeLa cells were used and cultured as described [65].

The 2,854-bp DNA fragment of the rat *Nis* promoter (pNIS-2.8) which contains the NUE region with two Pax8 binding sites was cloned in our laboratory [17]. Full length Pax8, Sp1, and CTCF were subcloned respectively in pcDNA3.1+, pBS and pcDNA1 Neo, and have been previously described [66-68].

### Chromatin immunoprecipitation

ChIP samples were prepared from PCCl3 cells as follows: cultures of 10 × 10^6^ cells were cross-linked with 1% formaldehyde for 10 minutes at room temperature. Cross-linking was stopped by the addition of glycine to a final concentration of 125 mM, and cells were washed twice with PBS. The cell pellet was resuspended consecutively in ChIP lysis buffers [69] and sonicated for 90 minutes (30 seconds high frequency pulsing followed by 30 seconds resting) using the Bioruptor sonicator (Diagenode, Denville, NJ) to produce chromatin fragments of 200–500 bp on average. After isolating the sheared chromatin, we incubated it with Pax8 antibody-coated magnetic beads. To prepare these beads, 100 μl of magnetic sheep anti-rabbit IgG beads (Invitrogen, Carlsbad, CA) were incubated overnight with 10 μg polyclonal anti-mouse Pax8 antibody (Biopat, Milan, Italy), that recognizes also rat Pax8 at 4°C. The following day, the beads were rinsed and added to the sheared chromatin and incubated overnight at 4°C. Samples were then rinsed five times with RIPA buffer, and the antibody was stripped from the beads by incubating in 1% SDS at 65°C for 15 minutes; cross-linking was reversed by incubating overnight at 65°C. The next day, samples were sequentially treated with RNAse A and Proteinase K, phenol-chloroform extracted, ethanol precipitated in the presence of 20 μg glycogen, and resuspended in 50 μl 10 mM Tris pH 8.0. Procedure controls included an input condition, obtained before DNA-protein complex sonication and further used during ChIP-Seq assays as normalization sample, and non-immunoprecipitated DNA (non-IP DNA), which was obtained just prior to Pax8 immunoprecipitation.

Before sequencing, Pax8-IP DNA (IP) was used to confirm enrichment of target DNA fragments (Additional file 13) by means of real time-PCR, using as positive IP controls both the *Nis* upstream enhancer element (NUE) and *Tpo* promoter sequences [7,9]. Negative controls of Pax8 binding to genomic DNA included promoter areas of *Gad1* (glutamate decarboxylase 1) and *Afm* (afamin or alpha-albumin), and a region of the *Nis* locus that does not bind Pax8. PCR reactions were assembled in triplicate with SYBR Green ER qPCR Supermix (Invitrogen, Carlsbad, CA) and run on an Applied Biosystem 7500 Real Time PCR system. The enrichment of target sequences in ChIP material was calculated relative to the *Afm* negative control, and normalized to their relative amplification in non-IP DNA.

### Illumina high-throughput sequencing

After verifying Pax8 target enrichment, IP and non-IP DNAs were modified for sequencing following the ChIP-Seq manufacturer’s protocol (Illumina, San Diego, CA). Briefly, DNAs were blunted with a combination of T4 DNA polymerase, Klenow polymerase, and T4 PNK. Then, a single 3′-end “A” base was added using Klenow exo (3′-to-5′ exo minus). Adapters provided by Illumina were ligated to the ends of the modified DNA before size selection of 200-bp fragments via polyacrylamide gel electrophoresis followed by extraction. The isolated DNA samples were used as the template for amplification by 18 cycles of PCR, and used for cluster generation on the Illumina Genome Analyzer II. Amplified products were column-purified with the QIAquick PCR Purification Kit (Qiagen, Dusseldorf, Germany) and assayed for quantity and quality with the Agilent 2100 Bioanalyzer (Agilent Technologies, Santa Clara, CA).

The 50 bp sequence reads were aligned to the rat genome (rn4; NCBI build 4) using the MIRO pipeline (Centre for Genomic Regulation, Barcelona, Spain) allowing 2 mismatches, and aligned tags were converted to BED format and used for identification of binding sites. In order to visualize the data in the University of California Santa Cruz genome browser (http://genome.ucsc.edu), the sequence reads were directionally extended to 300 bp, and for each base pair in the genome the number of overlapping sequence reads was determined and averaged over a 10 bp window. All sequencing data can be downloaded from Gene Expression Omnibus (GEO) under accession number GSE26938.

### Peak finding and data analysis

MACS program (Model-based Analysis for ChIP-Seq, v.1.4.1) was used with default parameters to determine enriched Pax8 binding regions using non-IP DNA as control. PeakAnalyzer software [70] was assessed to identify functional elements proximal to the immunoprecipitated peaks using the annotation of the Ensembl release 63 [71] on the RGSC genome assembly v3.4.

The distance correlation analysis was done with the GenomeInspector tool of the Genomatix suite (Genomatix Software GmbH, Munich, Germany), within +/−10Kb from the middle of the CpG islands and simple nucleotide repeats. CpG islands and simple nucleotide repeat coordinates were obtained from the UCSC Genome Browser [72].

### Motif search

We used two approaches for motif search in the Pax8 binding sites defined by MACS. The first method, based on MEME-ChIP [73] and focusing on the central 100 bp portion of each sequence, was used for the 500 peaks with the best FDR. The TOMTOM program was used to compare known transcriptional motifs with the motifs identified by MEME-ChIP [74]. On the other hand, the RegionMiner tool of the Genomatix suite (Genomatix Software GmbH, Munich, Germany) identified the most over-represented motifs, based on the background of occurrences of the transcription factor binding sites (TFBSs) within the whole sequence of the rat genome (rn4; NCBI build 4); we compared these to the Pax8 binding regions identified by MACS. The motifs were ranked by the Z-score/fold change to obtain the most relevant sites [15].

### Coimmunoprecipitation assays

Polyclonal antibodies (1 μg) were bound to Dynabeads (Invitrogen, Carlsbad, CA) and incubated with 200 μg of nuclear proteins extracted as described [75] from PCCl3 thyroid cells. The incubation was performed in 300ul of Immunoprecipitation (IP) buffer (20 mM HEPES pH 8, 10 mM KCl, 0,15 mM EGTA, 0,15 mM EDTA, 150 mM NaCl, 0.1% NP-40 with a cocktail of protease inhibitors (Roche, Manhein Germany)). After washing with IP buffer, proteins were eluted in 20 μl of Laemmli sample buffer and boiled for 10 minutes. The immunocomplexes were analyzed by SDS-PAGE and then immunoblotted using anti-Pax8 (BioPat, Milan Italy) and anti-Sp1 (Santa Cruz Biotechnology Inc., Santa Cruz, CA) antibodies. In the case of CTCF and prior to CoIP, we transfected into PCCl3 cells an expression vector containing the full-length human CTCF, as the antibody used (Upstate Biotechnology, Waltham, MASS) recognized the human form more specifically.

### Promoter activity assays

HeLa cells were transiently transfected using calcium phosphate with 1 μg of pNIS-2.8 reporter alone or in combination with 0.5 μg of expression vectors for Pax8, Sp1 and CTCF as indicated in the text. The Renilla luciferase-encoding pRL-CMV vector (50 ng) was used to correct for transfection efficiency. Forty-eight hours after transfection cells were harvested, lysed, and analyzed for firefly and renilla luciferase activities by the Dual-Luciferase reporter assay system (Promega, Madison WI). Promoter activity was determined as the ratio between firefly and renilla luciferase and represented as relative luciferase activity. The results were expressed as the mean ± SD of three independent experiments, each performed in triplicate. Data were analyzed with GraphPad Prism (Intuitive Software for Science, San Diego, CA). Statistical significance was determined using an Anova one-way test, and differences were considered significant at a *P* < 0.05.

### Expression arrays

The Pax8-dependent gene expression study was performed in differentiated PCCl3 thyroid cells by means of expression arrays (Agilent rat whole genome 44 K arrays). For this purpose, we generated three different conditions to finally establish two main comparisons: wild type vs. Pax8-silenced PCCl3 cells (si*Pax8* PCCl3), and scrambled siRNA-treated (siScramble PCCl3) vs. Pax8-silenced PCCl3 cells. Given that each comparison was performed using quadruplicates and dye-swaps (Cy3 and Cy5 fluorochromes), our experimental design included sixteen independent competitive hybridizations (Additional file 14).

Transient transfections of PCCl3 cells were performed using Lipofectamine 2000 (Invitrogen, Carlsbad, CA), both for scrambled and for *Pax8* siRNA conditions (10 ng siRNA /ml) (Dharmacon, Denver, USA). Pax8 silencing was tested by means of western blotting using a polyclonal Pax8 mouse antibody (Biopat, Milan, Italy) at different time points (24 and 48 hours) after transfection (Additional file 15). Once the 48 hours condition was defined as the best time point for Pax8 silencing, we performed additional transfections to isolate total RNA using TRIzol reagent (Invitrogen, Carlsbad, CA) for each condition considered (si*Pax8*, scrambled siRNA and PCCl3 cells treated with lipofectamine) following the manufacturer’s recommended protocol. RNA quality was evaluated with the Agilent 2100 Bioanalyzer and later amplified and labelled by using the Low RNA Input Linear Amplification Kit PLUS, Two-Color (Agilent Technologies, Palo Alto, CA). Briefly, for each sample 2 μg of total input RNA were amplified in two rounds of amplification following the manufacturer’s instructions. First strand cDNA synthesis and amplification reactions were carried out using random and T7 primers, respectively. During the 2-hour *in vitro* transcription, Cy3- or Cy5-labeled CTP was incorporated into each amplified RNA (cRNA). Products of the reaction were then purified using RNAeasy mini spin columns (Qiagen, Dusseldorf, Germany). Hybridization and slide and image processing were carried out according to the manufacturer’s instructions (Two-Color Microarray-Based Gene Expression Analysis protocol). In each experiment, 825 ng of contrasting cRNA samples were fragmented at 60°C for 30 min and hybridized at 65°C for 17 hr. The slides were scanned at a 10 μm resolution using the Agilent G2565BA Microarray Scanner (Agilent Technologies, Palo Alto, CA). Signal quantification was carried out with Feature Extraction 9.1 software (Agilent Technologies, Palo Alto, CA), using default analysis parameters for Agilent’s whole rat genome 44 K gene expression arrays. Array data were normalized using loess and quantile methods for normalization within and between arrays, respectively. Differential expression analysis was done using Bioconductor’s limma package. At a later stage, we used the annotate package and the data base rgug4131a.db to obtain the annotations of the rat genome from Agilent. Genes that showed adjusted *p*-values <0.005 were considered differentially expressed both in wild type vs. si*Pax8* cells and in siScramble PCCl3 vs. si*Pax8* PCCl3 cells. Functional analysis of Gene Ontology (GO) terms was carried out using the FatiGO tool and gene set enrichment analysis was performed using FatiScan [76,77]. All microarray data can be downloaded from the Gene Expression Omnibus (GEO) under accession number GSE26938.

### Experimental validation for ChIP-seq and expression array data

Technical validations were performed by means of real-time PCR to verify the Pax8-dependency of 7 *loci*, which showed significant results for both expression profiling and ChIP-Seq, independently of the peak location along the considered gene. After performing an independent Pax8-chromatin IP, we obtained genomic DNA from IP, non-IP, and input samples, which were further used to amplify specific fragments contained in IP peaks (Additional file 13). The immunoprecipitation ratio for IP peaks was estimated comparing IP versus non-IP amplification values using as normalizing regions those mentioned above (*Gad1* and *Afm*), which were confirmed as negative controls by the ChIP-Seq results.

siRNA transfection was done to obtain cDNA for each of the three conditions initially considered for expression profiling (*Pax8* siRNA, scrambled siRNA, and wild type PCCl3 cells). Expression level changes were defined in 7 down-regulated genes and in 4 up-regulated genes by means of real-time PCR for fragments specifically amplifying transcripts of interest (Additional file 16), using GAPDH as a control for target gene expression normalization.

## Abbreviations

TFs: transcription factors; ChIP-Seq: Chromatin immunoprecipitation followed by massive sequencing; IP: immunoprecipitated sample; Non-IP: non-immunoprecipitated sample; MACS: Model-based Analysis for ChIP-Seq; CpG: CpG island; GO: Gene Ontology.

## Competing interests

The authors declare that they have no competing interests.

## Authors’ contributions

SRL conceived, designed, and carried out experiments and wrote the manuscript. ECSP designed and carried out analyses and wrote the manuscript. ASP carried out coimmunoprecipitations, luciferase and qRT-PCR assays. CMC carried out analysis of microarray data. GGRP assisted in bio-informatics analysis including peaks mapping. JF participated in manuscript preparation. AV participated in manuscript preparation. DGP supervised and participated in manuscript preparation. PS supervised, participated in manuscript preparation and wrote the manuscript.

## Public database accesion number

Sequencing and gene expression microarray data have been deposited in the GEO database (accession number GSE26938).

http://www.ncbi.nlm.nih.gov/geo/query/acc.cgi?token=tjwrbocecmewahm&acc=GSE26938

## Supplementary Material

Additional file 1 (A). Pax8 chromatin immunoprecipitation validation performed to confirm enrichment of target DNA fragments by means of real-time PCR. Sequences belonging to the *Nis* upstream enhancer element (NUE) [7] and *Tpo* promoter sequences [9], previously described as Pax8 binding sites in rat thyroid cells, were used as positive controls. (B). ChIP-Seq results with regard to the *Nis locus* were visualized in the UCSC genome browser. Significant immunoprecipitated peak corresponding to MACS program included the *Nis* upstream enhancer (NUE), previously described to be regulated by Pax8 (underlined red letters). Click here for file

Additional file 2 **ChIP-Seq MACS data:** Excel file containing genomic coordinates of 13,151 rat genomic regions significantly immunoprecipitated according to the MACS ChIP program. Click here for file

Additional file 3 **ChIP seq peaks used for MEME/TOMTOM consensus motif analysis:** Genomic coordinates of the 500 most significant ChIP peaks used to verify Pax8-dependent immunoprecipitation and to delineate the consensus Pax8 DNA binding site. Click here for file

Additional file 4 **DNA binding motifs overrepresented in Pax8 IP peaks.** Genomatix matrices significantly associated to Pax8 MACS peaks. *Overrepresentation (genome)* column represents overrepresentation values of these matrices in our IP peaks compared to their presence along the rat genome, and *Z-Score (genome)* column indicates association value of Pax8-immunoprecipitated DNA for each considered DNA matrix. Click here for file

Additional file 5: **Expression data of wt and scrambled conditions vs. siPax8 conditions and association with ChIP-Seq data.***Common probes (+/− 1kb)* Excel sheet includes expression data for the 78 probes common to both expression comparisons (wt and siScramble conditions vs. si*Pax8; p*<0.005) and belonging to genes showing a significant IP peak within 1kb of a TSS. Additional Excel sheets include significant probes for each array comparison. Click here for file

Additional file 6 **FatiScan gene set enrichment analysis.** Excel file containing Gene Ontology (GO) terms commonly overrepresented in both expression array comparisons (datasheet “Common GO terms") and their adjusted *p*-values. This file also contains datasheets showing GO terms statistically significant for each individual comparison (WT or SCR sign GO biol. process). Click here for file

Additional file 7 **FatiScan gene set enrichment analysis for scrambled and wild type conditions vs. siPax8 conditions.** FatiScan image showing enriched GO terms in siScramble vs. siRNA*Pax8* and wt vs. siRNA*Pax8* comparisons, respectively. Click here for file

Additional file 8 **FatiScan gene set enrichment analysis for scrambled and wild type conditions vs. siPax8 conditions.** FatiScan image showing enriched GO terms in siScramble vs. siRNA*Pax8* and wt vs. siRNA*Pax8* comparisons, respectively. Click here for file

Additional file 9 **Significant biological processes among underexpressed probes.** FatiGO images showing overrepresented biological processes among common downregulated (n=633) probes for both expression array comparisons. Click here for file

Additional file 10 **Significant biological processes among overexpressed probes.** FatiGO images showing overrepresented biological processes among common upregulated (n=565) probes for both expression array comparisons. Click here for file

Additional file 11 **Overrepresented KEGG pathways among under- and overexpressed probes.** KEGG pathways enriched among common downregulated (n=633) and upregulated (n=565) probes for both expression array comparisons. Click here for file

Additional file 12 UCSC genome browser images showing significant Pax8 IP peaks for closely positioned *loci* which were detected to be significantly deregulated in expression arrays (p<0.005). Click here for file

Additional file 13 Oligonucleotides used for immunoprecipitation validation prior to performing high throughput sequencing (including positive and negative Pax8 immunoprecipitation controls), or for experimental validation of ChIP-Seq. Click here for file

Additional file 14 Schematic representation of experimental design followed for whole genome rat expression arrays. Both comparisons (PCCl3-si*Pax8* vs. PCCl3-wt and PCCl3-si*Pax8* vs. PCCl3-siScramble) included four different biological replicates that were cross-labelled with either Cy3 or Cy5. Click here for file

Additional file 15 Immunoblot demonstrating Pax8 downregulation in si*Pax8* conditions (si*Pax8*) versus control conditions, including the no transfection condition (wt) and siScramble PCCl3-transfected cells (siScramble). Time points include 24 and 48 hours. Click here for file

Additional file 16 Oligonucleotides used for experimental validation of expression arrays. Click here for file
