# Supplementary material for: Genome-wide analysis of Pax8 binding provides new insights into thyroid functions
Source: BMC Genomics. 2012 Apr 24;13:147. doi: 10.1186/1471-2164-13-147 (PMC3403905; doi:10.1186/1471-2164-13-147)

A

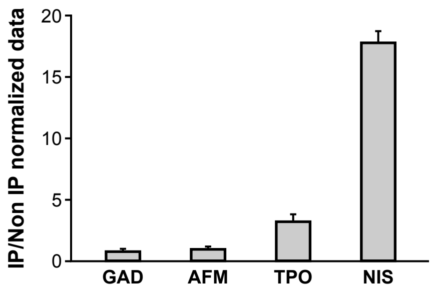

B

```
>rn4_ct_MACS2011
range=chr16:19043136-19045861
TTCGGGAGTACAAGGTGGTGGGGCGTTGCTTGCCAACCCCAAAATGCCACACACCGCCACTGTACCGAATGCGAATC
TTTGCACCCAACCATGTGGTGGCCAAGTCCCGCTTCTGGTACTTTGTGTCGCAGCTGAAGAAGATGAAGAAGTCATC
CGGGGAAATTGTGTACTGTGGGCAGGTGAGCCTTGGGGCCCCACCGGATGACGGTCCCTGGGTACCATCAGAGCCAG
GGCAGTACACAAGCCTCACATCCTCCTTACTCCCCACAGGTGTTTGAGAAGTCACCCCTGCGTGTGAAGAACTTCGG
CATCTGGCTGCGCTATGATTCCCGAAGTGGCACTCACAACATGTACCGAGAGTACCGGGACCTGACCACTGCCGGCG
CGGTCACACAGTGCTGTGAGTCACCAACCCTCTGTCAACCCTTGGCACCCAACCCAAGAACTGCCGCTACTATAGCG
GCTGTTTAAACAGTGGTGCACACAGCAAAGTGCTTCGTCGCCTTTGGGGGGCTGTGCAGTGCCCTCCAGAGTGAATC
AGGAGGTTCTACAGTTCTGTGGGTGTTCTGGGTGGCTGGGGCTGACTTTCTTCTCTTTCCACAGACCGAGACATGGG
TGCCCGACACCGTGCCCGTGCGCACTCCATCCAGATCATGAAGGTGGAAGAGATTGCAGCTGGCAAGTGCCGCCGGC
CAGCTGTCAAGCAGTTCCACGTGAGTGAACCTGAGTGTGGGGCTGACGCAGGGCTGCAGGGGGTGGTTGCGCATGGA
CAGTCCCTGGACATGCATCTCCTCCCTCCCCACAGGACTCCAAGATCAAGTTCCCATTGCCCCACCGTGTGTTGCGG
CGCCAGCACAAACCACGCTTCACCACCAAGAGGCCAAACACCTTCTTCTAGACACAGAGACCCACTGAATAAAAGCT
TCAGACTGTCCTTGCTTGTTTGCCTCTGTGTCCCTGACAGGGTCCCAAGTGGACCCTAACAACAACAGGCTTGCCCC
ACCTGCCTGCCAGCCTTGGTTCAGTGGCATCTTTCTATAGTTGTGTATCCTACTGAGTTAACTCAACCCCACTGCAG
TTTGTGTGGGACATAGCCTGAGCCCTGTGTGTGACACCGAAGGGTGGCAGGCTGTTTCTGCAGTCTCCAGGGTGCTG
GCTTGGGCCTGAGAATTCCAGACATGAACATAATCCAGGCCTGGGGACCTGGTTGAGCTCTTTATCCTGCTGGCTCT
AAGCCTCTGCTAGGTGGGAATGAGGCCAGCCAAACTCCGGGGACCTTGAGCCACCAAGCTTTAGGTGAGGACTACAC
TGTGGATCATGGAAACACGGGGTTGCTTTTGTTGGGTGTGGCCAGCACCATCACGGTGCCTGGTTTTGAAGGAGTAT
AGCCTGAAGCCTCCGTGGCTGTCTTGTCACTGCTCTCAAGTCTCTACTGGCCACAGAGAGGAACACTGCACTTGCTT
CTAGCCCCTTGCAGATATTTCCATACGTGTATGCACATAGCCCAGGGTAATTTTGAGTTTCAACCATCCAGTGCCAG
CTAGAGTAAAAGCCTGTGTCTATACTATGAAGGCCATAAGAGGGCACCGTGTCGAAGAGAAGTCTTCCCACTGAGTC
CATTGGCCCCACATCAAAGGAATGGGTGATCCCTCTGACTATGCAGTGTTTCCCTGTGCATTGTGGGGCCAGTGGCT
GCTACTTTAAAGTCATGAGGAATCTGGACACTCTGAGGACCTGGGTTTGTCCCTGTATTAAGATACTGCTCCAGGGC
ATCGGATGCCACTGGCCTCTGCAGGCACCCGTATTCATGCAACTATCCTTCACCCACATACATAATTGTGGTTTTTT
TGGGAGTTTGGGGAAATGATGTGTGTGACTAAGCCACTAGGAGTCAAAAGTGTAGGCCATTCTGGTCTACAAGGTGA
GTTCCAGAACAGCCAGGGCTACACGGAGAGGCCCTATGACATAAAGTTGCAGTGGCATGCAACACCAGCTCCAGCCC
TCCCTGGTGGCTGAGCACCGGCATGACCGCGAGTGTCCCAGCATGGCAATCCAGCCTACCTGCCTCTGGAAGGCTCT
TCCCCTCCCTCCTGTTTGTCGACCTCTTAGCCTGCCACCTAGGCTTACGAGCCTGCCCTAGTCAGGTCTCCTGACAG
CACTTCCAGGCGGGCGGCGAGTCCATATACATCATGAGCCCACAAGGGGGCGCGTGCCTTAGTCCTACCTGCCAGCC
ACCAGAAACGGAGGACTCCACAGGCAGTCAACCCTGAACACCCCAGAACCGGCCAGTTCACCAGAAAAGGGTCCCTT
GAAAGTCACAATGGGCAAACTGTTCTCACCCAGTTATGCCTGCGATAGAAGGGAGTGATGGGTGCCCAACAAACCCT
CACTTCTCATCCCAGCAGTAAGGAAGCTAAGGCAGAAGGACCTCACCTCCGAGGCCAGCCTGTGGGAAAGGGAAAAC
TGAGAAGACAGCCAGGTCAGGACAACATGCCAGCCTGAGCTGCATGAGACTTAGCCTCAAACGCGTGTGCACATGTA
CACACATCATAGGTCCAGAGTGCCCTTGTACAGGTGGGGAGTCCTAAAGCAGAAGAAAGAAATTCTCCAAGAGAACC
TGAGTGCCTCCCACCGGGTTAGCTGAAGGGCAAGGTTGGACAGTTCCTTCTCCCAAGCTGCGGAGAAAGGTAGATGC
TCTCTGGGGGAAGGGCTCCAGTCGCTCGCTT
```

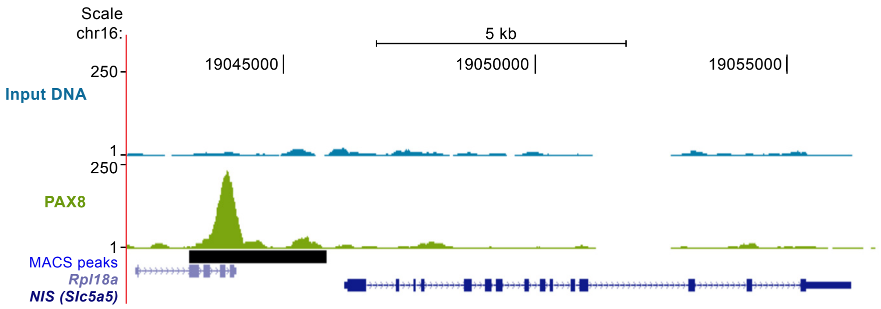

Supplement: Additional file 1 — (A). Pax8 chromatin immunoprecipitation validation performed to confirm enrichment of target DNA fragments by means of real-time PCR. Sequences belonging to the Nis upstream enhancer element (NUE) [7] and Tpo promoter sequences [9], previously described as Pax8 binding sites in rat thyroid cells, were used as positive controls. (B). ChIP-Seq results with regard to the Nis locus were visualized in the UCSC genome browser. Significant immunoprecipitated peak corresponding to MACS program included the Nis upstream enhancer (NUE), previously described to be regulated by Pax8 (underlined red letters). [file 1471-2164-13-147-S1.pdf]
