# Supplementary material for: Genome-wide analysis of Pax8 binding provides new insights into thyroid functions
Source: BMC Genomics. 2012 Apr 24;13:147. doi: 10.1186/1471-2164-13-147 (PMC3403905; doi:10.1186/1471-2164-13-147)
Supplement: Additional file 3 — ChIP seq peaks used for MEME/TOMTOM consensus motif analysis: Genomic coordinates of the 500 most significant ChIP peaks used to verify Pax8-dependent immunoprecipitation and to delineate the consensus Pax8 DNA binding site. [file 1471-2164-13-147-S3.doc]

chr1 131476527 131477033

chr1 135835147 135835745

chr1 135843615 135844597

chr1 154053931 154054545

chr1 154062347 154064218

chr1 156007864 156008551

chr1 156149969 156151275

chr1 156766763 156767669

chr1 157142370 157143240

chr1 15892533 15893506

chr1 172046331 172046859

chr1 172069520 172070055

chr1 174118584 174119563

chr1 176583415 176583737

chr1 184596425 184596779

chr1 191290836 191291732

chr1 195198819 195199201

chr1 197237581 197238075

chr1 200959343 200959691

chr1 20422128 20422675

chr1 205031939 205032490

chr1 205807799 205808462

chr1 208201800 208202494

chr1 208447027 208448406

chr1 212386436 212387447

chr1 213211033 213211786

chr1 219279041 219279880

chr1 222360914 222361408

chr1 222361596 222362036

chr1 227426605 227427657

chr1 227909915 227910494

chr1 22816291 22816583

chr1 228474710 228475292

chr1 228622159 228622569

chr1 232683438 232684595

chr1 236154113 236154834

chr1 236295043 236295442

chr1 236329495 236329930

chr1 238896138 238896449

chr1 245451922 245452473

chr1 248657771 248659177

chr1 249884409 249885492

chr1 249900959 249901960

chr1 250696236 250696985

chr1 251679347 251680071

chr1 252299174 252299589

chr1 252682360 252683766

chr1 252721463 252722642

chr1 261456345 261457749

chr1 262671423 262672582

chr1 262948176 262949003

chr1 263165933 263166636

chr1 263714751 263716269

chr1 266246460 266247212

chr1 43163627 43164791

chr1 45179099 45179682

chr1 45494338 45494985

chr1 4584421 4585033

chr1 53263177 53264302

chr1 54104169 54105581

chr1 54734872 54735405

chr1 78440107 78440894

chr1 85982151 85983360

chr1 85990294 85992720

chr1 90919744 90920147

chr1 95050081 95051032

chr1 96189153 96190255

chr1 96237691 96238180

chr1 99294526 99295992

chr10 102647334 102648284

chr10 107478470 107480545

chr10 109569254 109570270

chr10 109766555 109767467

chr10 110408879 110410793

chr10 13102007 13102864

chr10 16369134 16369566

chr10 16426140 16428311

chr10 16827721 16828668

chr10 16878705 16879983

chr10 16926693 16927239

chr10 19593487 19593904

chr10 29850034 29850783

chr10 30974170 30975204

chr10 31302168 31303464

chr10 31424165 31424644

chr10 3602464 3603054

chr10 36883591 36883927

chr10 38993189 38994411

chr10 4156086 4156965

chr10 43279516 43280826

chr10 44950417 44950809

chr10 45367322 45368874

chr10 46081164 46081816

chr10 46220077 46221320

chr10 5104852 5105747

chr10 5752607 5752956

chr10 63210195 63211794

chr10 69020967 69021440

chr10 72943955 72944640

chr10 73962978 73963627

chr10 76041429 76043132

chr10 76842179 76842966

chr10 77323863 77324305

chr10 78357171 78358330

chr10 83762704 83764135

chr10 83864530 83866217

chr10 90094123 90095785

chr10 91016566 91017250

chr10 91503780 91504957

chr10 93295233 93296149

chr10 95967425 95968148

chr10 96113831 96114718

chr10 96640476 96640983

chr10 98526730 98527466

chr11 31400992 31401793

chr11 31577457 31578491

chr11 36998831 36999191

chr11 37967066 37967560

chr11 57720284 57721172

chr11 63593166 63593457

chr11 67491451 67491999

chr11 71644699 71645989

chr11 71890373 71891373

chr11 76362300 76362952

chr11 77875112 77876372

chr11 78245066 78245456

chr11 79843924 79844969

chr11 81649406 81651903

chr11 82591213 82592743

chr11 85228669 85229314

chr12 16362736 16363631

chr12 23736391 23737703

chr12 28221686 28222305

chr12 28886494 28887679

chr12 33569420 33570270

chr12 34698797 34700588

chr12 3561429 3561862

chr12 35728026 35729694

chr12 35890738 35891965

chr12 36741848 36742897

chr12 38981003 38981863

chr12 39433458 39434299

chr12 42234425 42235533

chr12 6297248 6298493

chr12 6975434 6975788

chr12 9190858 9191118

chr12 9774813 9775522

chr13 101177836 101178262

chr13 101211824 101212190

chr13 103458775 103459634

chr13 103577806 103578720

chr13 107501010 107502180

chr13 12880080 12880749

chr13 30943806 30944737

chr13 31412708 31413651

chr13 31633010 31634021

chr13 31670412 31671157

chr13 66857497 66858124

chr13 68725782 68726495

chr13 97482052 97482664

chr14 102017471 102017880

chr14 102161891 102162550

chr14 12887094 12887591

chr14 22017734 22018061

chr14 36430020 36431021

chr14 44577481 44578642

chr14 62716508 62717235

chr14 67517491 67518140

chr14 80071525 80072879

chr14 80950400 80951839

chr14 81073402 81074242

chr14 81378143 81380041

chr14 85941645 85942429

chr14 86497644 86498556

chr14 93149473 93150974

chr15 22434526 22434854

chr15 22747389 22748345

chr15 2465764 2466121

chr15 32440724 32441743

chr15 3623469 3624111

chr15 39765132 39765520

chr15 40749381 40749756

chr15 41354042 41354406

chr15 4341013 4342531

chr15 4395341 4398411

chr15 51263628 51264235

chr15 54117558 54118789

chr15 54121979 54122330

chr16 10625347 10626326

chr16 19043135 19045861

chr16 19215368 19217524

chr16 46896719 46897070

chr16 47628496 47629415

chr16 5155422 5155920

chr16 5471596 5472116

chr16 60546530 60546888

chr16 60818834 60819289

chr16 61494229 61494543

chr16 65014105 65014541

chr16 70880026 70880530

chr16 72640324 72640686

chr16 74604783 74605177

chr16 75746887 75747315

chr16 79563355 79564054

chr16 79643407 79644269

chr16 81752490 81754161

chr16 83820690 83821355

chr16 83956777 83957232

chr17 10050131 10050986

chr17 11555974 11556373

chr17 14456679 14458374

chr17 14476461 14477409

chr17 15696324 15699094

chr17 24677327 24677991

chr17 25833592 25834204

chr17 25996050 25996764

chr17 27024892 27025297

chr17 29903150 29903554

chr17 33152936 33153528

chr17 48175188 48175571

chr17 63850171 63850601

chr17 7566095 7566790

chr17 93041159 93041889

chr18 29353348 29353995

chr18 3254586 3255005

chr18 52517120 52517977

chr18 54074466 54075068

chr18 56680599 56681497

chr18 56736865 56737794

chr18 61398885 61400005

chr18 72040681 72041391

chr18 72533432 72534174

chr18 75096906 75097719

chr19 14273699 14275007

chr19 21093866 21094178

chr19 26245404 26245932

chr19 45274452 45275360

chr19 47308615 47309520

chr19 47451219 47452325

chr19 477213 477924

chr19 50422706 50423761

chr19 50873472 50875429

chr19 51043860 51044967

chr19 51128740 51129510

chr19 52879862 52880889

chr19 54087345 54087872

chr19 54776563 54777221

chr19 54783135 54784106

chr19 56762764 56764772

chr19 56844473 56844944

chr2 116622583 116623199

chr2 118766562 118766923

chr2 125014477 125015089

chr2 128472272 128472911

chr2 144304928 144305455

chr2 158486262 158486581

chr2 180342904 180344176

chr2 195383287 195384093

chr2 197473778 197474155

chr2 201080183 201080705

chr2 212523623 212524031

chr2 218155703 218156747

chr2 224994284 224995019

chr2 227050204 227050597

chr2 228743406 228743983

chr2 24379211 24379562

chr2 251154331 251154997

chr2 25886989 25887328

chr2 26902590 26902993

chr2 28604820 28605361

chr2 28656172 28656815

chr2 30395311 30396564

chr2 30452246 30452639

chr2 77212624 77213064

chr2 77382009 77382715

chr2 84746164 84746571

chr2 95198746 95199210

chr20 10225150 10226422

chr20 11111768 11112752

chr20 12013482 12014579

chr20 44491418 44492114

chr20 45716236 45716937

chr20 6518383 6519575

chr20 7975001 7975927

chr20 9376789 9377949

chr3 104925116 104926197

chr3 105721493 105722623

chr3 10596445 10597451

chr3 106044049 106045015

chr3 106408771 106409296

chr3 108590859 108591792

chr3 109123077 109123551

chr3 117468122 117469230

chr3 129319578 129319874

chr3 134377530 134378070

chr3 141261991 141262915

chr3 143641856 143642569

chr3 143815204 143816082

chr3 144724280 144724650

chr3 14759590 14760951

chr3 148887043 148888906

chr3 149301115 149301501

chr3 15329195 15330323

chr3 154818560 154819732

chr3 155444954 155446813

chr3 155630331 155631096

chr3 161544262 161544907

chr3 168671889 168673235

chr3 169148133 169148820

chr3 169743594 169744375

chr3 17643634 17644406

chr3 2658684 2659376

chr3 31561507 31562005

chr3 34380041 34380721

chr3 41124948 41125782

chr3 42485760 42486172

chr3 42734600 42734916

chr3 43206095 43206437

chr3 54695639 54696365

chr3 55543675 55545068

chr3 61540395 61540765

chr3 75745502 75745878

chr3 76306362 76307047

chr3 76883897 76884678

chr3 77674354 77675891

chr3 78388454 78389055

chr3 90819294 90819993

chr3 91066365 91067709

chr3 94731970 94732239

chr4 105281100 105281521

chr4 117081600 117082310

chr4 117084522 117084871

chr4 118432659 118433192

chr4 121423489 121424202

chr4 123261524 123262400

chr4 123300094 123301136

chr4 132303307 132303958

chr4 132602392 132602981

chr4 134910447 134911148

chr4 135230524 135231589

chr4 135343178 135344295

chr4 135833396 135833856

chr4 144022577 144023422

chr4 148873281 148873680

chr4 156423812 156424166

chr4 156796917 156797393

chr4 163747244 163747616

chr4 171953729 171954364

chr4 182838324 182839231

chr4 183912985 183913363

chr4 184359478 184359784

chr4 184511251 184511927

chr4 184753654 184755025

chr4 2461984 2462339

chr4 26029863 26030149

chr4 48159865 48160324

chr4 57333707 57334058

chr4 58069064 58069735

chr4 61089726 61090050

chr4 62730935 62732126

chr4 64787275 64788108

chr4 79610361 79610964

chr4 79987509 79987851

chr4 82579123 82579729

chr4 83123673 83124287

chr4 83729964 83730441

chr5 101306027 101306484

chr5 115361982 115362641

chr5 118429103 118429516

chr5 121804492 121804882

chr5 128386104 128386729

chr5 134779839 134781445

chr5 136439967 136440824

chr5 145609270 145610108

chr5 149160543 149161525

chr5 149357545 149358278

chr5 149359510 149359989

chr5 150924389 150925172

chr5 152968305 152969095

chr5 154398236 154399020

chr5 154804902 154806178

chr5 156421253 156421846

chr5 156817353 156817668

chr5 157187162 157187948

chr5 158043536 158044282

chr5 159473162 159474548

chr5 161095608 161096499

chr5 166017001 166017815

chr5 168254556 168255386

chr5 23405518 23405937

chr5 49394966 49395394

chr5 59317603 59318263

chr5 62669131 62670066

chr5 62809049 62809977

chr5 62811679 62813657

chr5 64701074 64702008

chr5 87619748 87620236

chr6 103022740 103023447

chr6 105497503 105498594

chr6 109184160 109184706

chr6 109244330 109245728

chr6 110487696 110488461

chr6 111013132 111015066

chr6 114646100 114646566

chr6 11670833 11671545

chr6 123535579 123536459

chr6 127495102 127496057

chr6 129853892 129854492

chr6 132827333 132828357

chr6 135660402 135661476

chr6 135674827 135675528

chr6 136844155 136844788

chr6 138020090 138021053

chr6 26787880 26789206

chr6 28637537 28638253

chr6 3305422 3305975

chr6 36268054 36268392

chr6 41322812 41323400

chr6 41701269 41701950

chr6 41876828 41877849

chr6 42158528 42159293

chr6 75047414 75049865

chr6 7639364 7639747

chr6 8867043 8868462

chr6 95649062 95649728

chr6 99167704 99170158

chr7 10102011 10104132

chr7 104531153 104533437

chr7 10545187 10547513

chr7 118859473 118859962

chr7 122260636 122261591

chr7 122295778 122296329

chr7 127623340 127624427

chr7 136549154 136549757

chr7 137441144 137442079

chr7 138338338 138339424

chr7 139400058 139400855

chr7 139476348 139477635

chr7 139790302 139790715

chr7 139906539 139907474

chr7 140122388 140123329

chr7 19745672 19746261

chr7 22784988 22785998

chr7 29013559 29013948

chr7 47110998 47111350

chr7 56065513 56066248

chr7 67496798 67498037

chr7 9685282 9685754

chr7 99825215 99825867

chr8 101879527 101880649

chr8 105997611 105998029

chr8 108492115 108492518

chr8 110806066 110806977

chr8 119355494 119356585

chr8 123480286 123481291

chr8 123619476 123620139

chr8 124244676 124245184

chr8 124600671 124601248

chr8 126086197 126087729

chr8 127109182 127110048

chr8 31736859 31737576

chr8 34784748 34785576

chr8 35114459 35115871

chr8 44920920 44921332

chr8 45278460 45278736

chr8 54245604 54246280

chr8 68209434 68210021

chr8 70354639 70354975

chr8 76143942 76145483

chr9 103366919 103367371

chr9 104379830 104380529

chr9 105491131 105491944

chr9 105575714 105576706

chr9 105855592 105857514

chr9 107232366 107233387

chr9 110952872 110953211

chr9 12119282 12120043

chr9 12410148 12411204

chr9 38395632 38396056

chr9 39203115 39203616

chr9 55685793 55686104

chr9 73995598 73996373

chr9 74765575 74766505

chr9 75209480 75210076

chr9 79917106 79917820

chr9 79956083 79956570

chr9 86056866 86057793

chr9 87144857 87145961

chr9 87607460 87608253

chr9 88447141 88448003

chr9 88981205 88982569

chr9 89600797 89601732

chr9 89783024 89784562

chr9 91681326 91682765

chr9 92958807 92959103

chr9 9619776 9620459

chrX 137827357 137827713

chrX 14631449 14631817

chrX 90519178 90519618
