# Supplementary figures and images for: Genome-wide analysis of Pax8 binding provides new insights into thyroid functions
Source: BMC Genomics. 2012 Apr 24;13:147. doi: 10.1186/1471-2164-13-147 (PMC3403905; doi:10.1186/1471-2164-13-147)

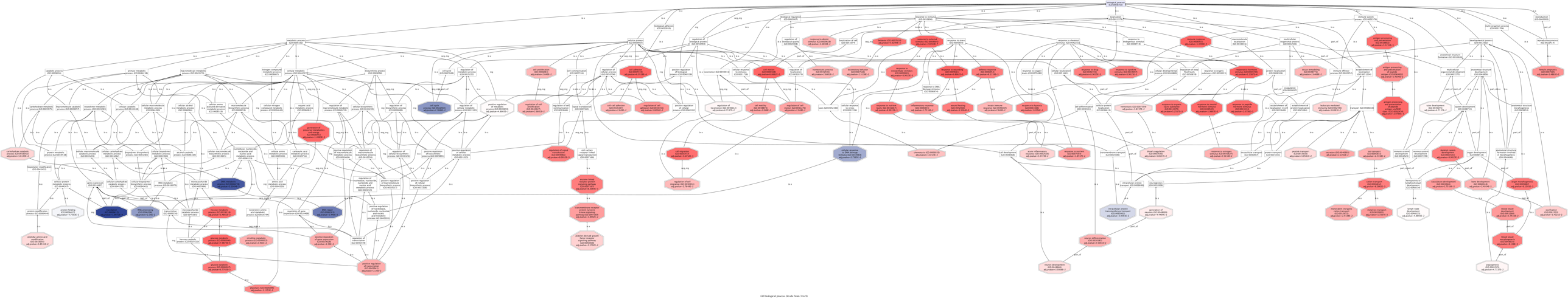

Supplement: Additional file 7 — FatiScan gene set enrichment analysis for scrambled and wild type conditions vs. siPax8 conditions. FatiScan image showing enriched GO terms in siScramble vs. siRNAPax8 and wt vs. siRNAPax8 comparisons, respectively. [file 1471-2164-13-147-S7.png]

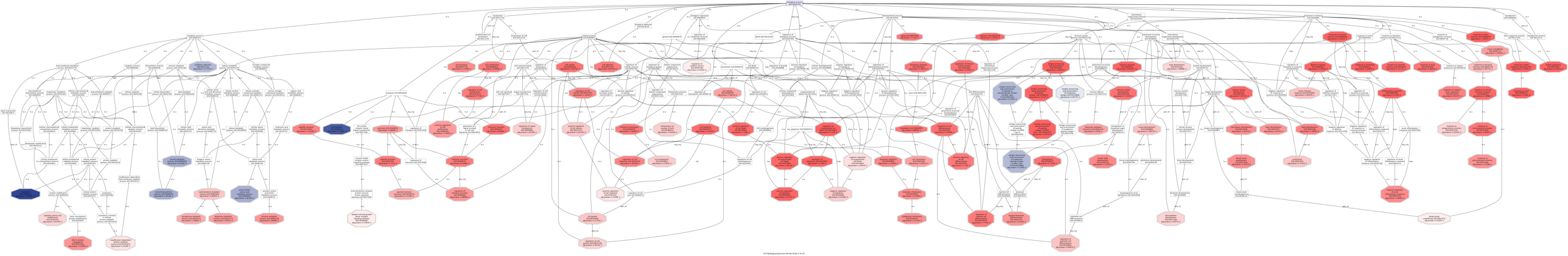

Supplement: Additional file 8 — FatiScan gene set enrichment analysis for scrambled and wild type conditions vs. siPax8 conditions. FatiScan image showing enriched GO terms in siScramble vs. siRNAPax8 and wt vs. siRNAPax8 comparisons, respectively. [file 1471-2164-13-147-S8.png]

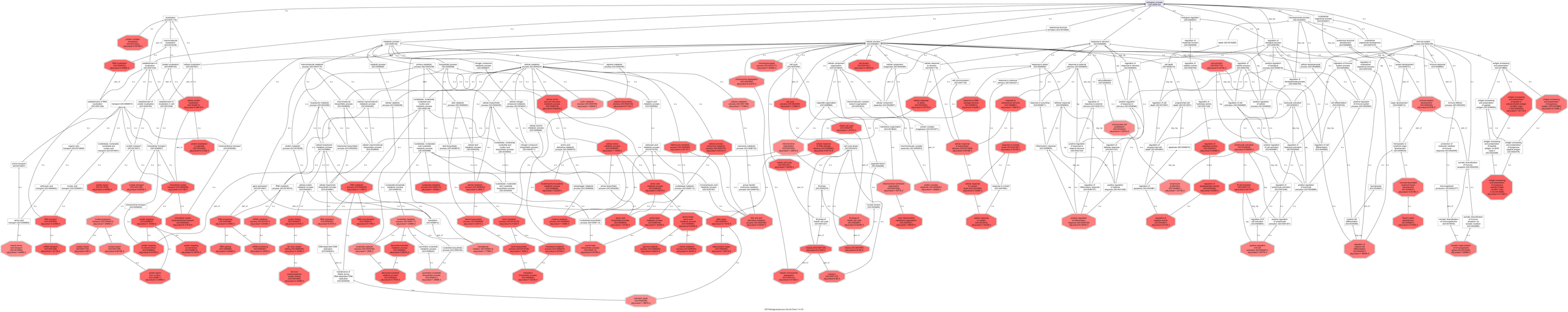

Supplement: Additional file 9 — Significant biological processes among underexpressed probes. FatiGO images showing overrepresented biological processes among common downregulated (n=633) probes for both expression array comparisons. [file 1471-2164-13-147-S9.png]

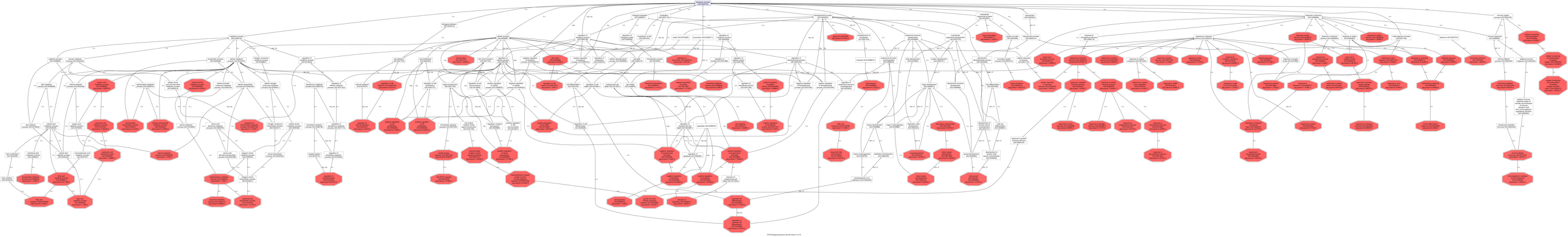

Supplement: Additional file 10 — Significant biological processes among overexpressed probes. FatiGO images showing overrepresented biological processes among common upregulated (n=565) probes for both expression array comparisons. [file 1471-2164-13-147-S10.png]

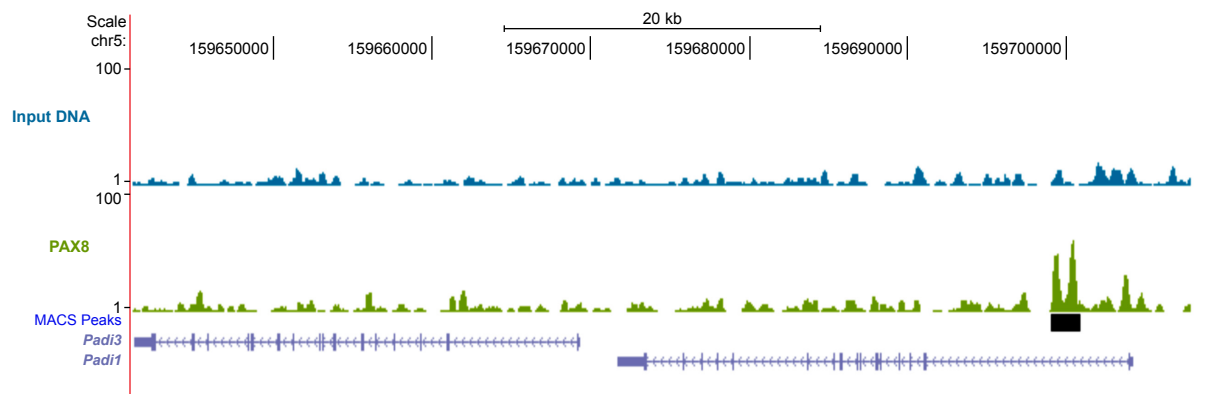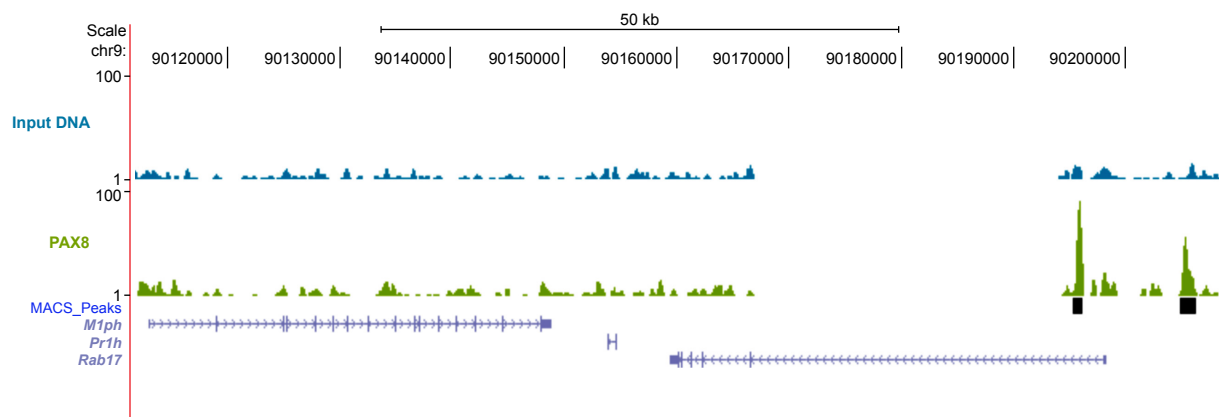

Supplement: Additional file 12 — UCSC genome browser images showing significant Pax8 IP peaks for closely positioned loci which were detected to be significantly deregulated in expression arrays (p<0.005). [file 1471-2164-13-147-S12.pdf]

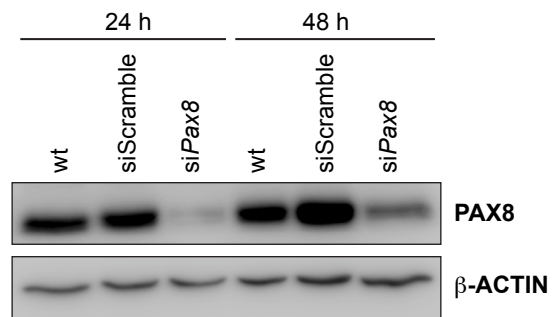

Supplement: Additional file 14 — Schematic representation of experimental design followed for whole genome rat expression arrays. Both comparisons (PCCl3-siPax8 vs. PCCl3-wt and PCCl3-siPax8 vs. PCCl3-siScramble) included four different biological replicates that were cross-labelled with either Cy3 or Cy5. [file 1471-2164-13-147-S14.pdf]
