# Supplementary material for: Genome-wide analysis of Pax8 binding provides new insights into thyroid functions
Source: BMC Genomics. 2012 Apr 24;13:147. doi: 10.1186/1471-2164-13-147 (PMC3403905; doi:10.1186/1471-2164-13-147)
Supplement: Additional file 13 — Oligonucleotides used for immunoprecipitation validation prior to performing high throughput sequencing (including positive and negative Pax8 immunoprecipitation controls), or for experimental validation of ChIP-Seq. [file 1471-2164-13-147-S13.doc]

| **Primer Name** | **Primer Sequence** |
| --- | --- |
| AFM f negative cont. | GCCTCTGGGACTTACTGCTG |
| AFM r negative cont | ATTAGAGCCAGAACGGCTGA |
| GAD1 f negative cont. | GTGTCTTCGAGCTCCCTCAC |
| GAD1 r negative cont | ATCCCAGGCCTGTCTCTTTT |
| NIS f negative cont. | TTTCCCTCCTGTCCCTTTTT |
| NIS r negative cont | GCCAGCCTGGTCTACAAGAG |
| TPO f | tgagtggcacctttgttctg |
| TPO r | CACTGAAGAAGCAGGCTGTG |
| NUE NIS f | TTCTCTTTCCACAGACCGAGACATGGGTGC |
| NUE NIS r | AGAGGCAAACAAGCAAGGACAGTCTGAAGC |
| DIO1 f | ACTTGGGCAAATGGATTGAG |
| DIO1 r | TTCAGTTCACAGCACCCAAC |
| BRCA1 f | GGACTCCCTCACACATCCAT |
| BRCA1 r | CGGAAGAAAGGTGAGACAGC |
| DAB2IP f | CTCTCTGAACCGCACAACAA |
| DAB2IP r | CCTTTTGGGAACAGTGGGTA |
| TMOD1 f | TGCTTCAGGTGACTTTGTGC |
| TMOD1 r | TGCCTCTTCCCAGAGTCTTC |
| LPP60 f | ATTATTCCCAGGGGGAACTG |
| LPP60 r | AGGACTGCTGTGGGATATGG |
| CDH16 f | GGGCAGTGACAGACCAAGAT |
| CDH16 r | TTCAGGAAGCTCTGGCAAAT |
| RAB17 f | ACCCTGATGCTTGTTGGAAG |
| RAB17 r | TTGACAGCTTTGCATGAACC |
| MYOVB f | AAGGCATGCTGGAGTACCAC |
| MYOVB r | GATGGAAGGGTTGGCTGTAA |

**ADDITIONAL FILE 13** Oligonucleotides used for immunoprecipitation validation prior to performing high throughput sequencing (including positive and negative Pax8 immunoprecipitation controls), or for experimental validation of ChIP-Seq.
