# Supplementary material for: Genome-wide analysis of Pax8 binding provides new insights into thyroid functions
Source: BMC Genomics. 2012 Apr 24;13:147. doi: 10.1186/1471-2164-13-147 (PMC3403905; doi:10.1186/1471-2164-13-147)
Supplement: Additional file 15 — Immunoblot demonstrating Pax8 downregulation in siPax8 conditions (siPax8) versus control conditions, including the no transfection condition (wt) and siScramble PCCl3-transfected cells (siScramble). Time points include 24 and 48 hours. [file 1471-2164-13-147-S15.pdf]

siRNA *Pax8* *vs.* siScramble

Pax8 siRNA PCCl3 cells sample 1<br>*Pax8* siRNA PCCl3 cells sample 2<br>*Pax8* siRNA PCCl3 cells sample 3<br>*Pax8* siRNA PCCl3 cells sample 4	*vs.*	Scramble siRNA PCCl3 cells sample 1<br>Scramble siRNA PCCl3 cells sample 2<br>Scramble siRNA PCCl3 cells sample 3<br>Scramble siRNA PCCl3 cells sample 4
Pax8 siRNA PCCl3 cells sample 1<br>*Pax8* siRNA PCCl3 cells sample 2<br>*Pax8* siRNA PCCl3 cells sample 3<br>*Pax8* siRNA PCCl3 cells sample 4	*vs.*	Scramble siRNA PCCl3 cells sample 1<br>Scramble siRNA PCCl3 cells sample 2<br>Scramble siRNA PCCl3 cells sample 3<br>Scramble siRNA PCCl3 cells sample 4

siRNA *Pax8* *vs.* Wt

Pax8 siRNA PCCl3 cells sample 1<br>*Pax8* siRNA PCCl3 cells sample 2<br>*Pax8* siRNA PCCl3 cells sample 3<br>*Pax8* siRNA PCCl3 cells sample 4	*vs.*	WT PCCl3 cells sample 1<br>WT PCCl3 cells sample 2<br>WT PCCl3 cells sample 3<br>WT PCCl3 cells sample 4
Pax8 siRNA PCCl3 cells sample 1<br>*Pax8* siRNA PCCl3 cells sample 2<br>*Pax8* siRNA PCCl3 cells sample 3<br>*Pax8* siRNA PCCl3 cells sample 4	*vs.*	WT PCCl3 cells sample 1<br>WT PCCl3 cells sample 2<br>WT PCCl3 cells sample 3<br>WT PCCl3 cells sample 4

Cy3 **Cy5**
