# Supplementary material for: Genome-wide analysis of Pax8 binding provides new insights into thyroid functions
Source: BMC Genomics. 2012 Apr 24;13:147. doi: 10.1186/1471-2164-13-147 (PMC3403905; doi:10.1186/1471-2164-13-147)
Supplement: Additional file 16 — Oligonucleotides used for experimental validation of expression arrays. [file 1471-2164-13-147-S16.doc]

| **Primer Name** | **Primer Sequence** |
| --- | --- |
| GAPDH f | CTCATGACCACAGTCCATGC |
| GAPDH r | TTCAGCTCTGGGATGACCTT |
| DIO1 f | TTTCATTCAAGGCAGCAGAC |
| DIO1 r | TTAAAAGCCCATCCATCTGTG |
| BRCA1 f | GATGGCTGCAGTGTGAGAGA |
| BRCA1 r | ATACGCGGACATTTTTCTGG |
| BRCA1 f | ACAGATCAGGAACAGCAATTTT |
| BRCA1 r | AAGGCCTTCTCATTCCTGACT |
| DAB2IP f | CACAGGCAAGGTGAAGGACT |
| DAB2IP r | GGGCTTTGATGAACTCACCT |
| TMOD1 f | GGCATTTGCTCTTGCTGAA |
| TMOD1 r | CTGGCTCTGGTTGTCGATTT |
| LPP60 f | CCTGAGGTCTGCCTGTTCTT |
| LPP60 r | TACCAGCTCACGGTTGATTG |
| CDH16 f | CTGGCAGCGATAGGTTTCAT |
| CDH16 r | GATCAGATGGAGCTGGGGTA |
| RAB17 f | AACACCATTGCCCAGGAG |
| RAB17 r | CAGGCTGAGCTATCCTCCAC |
| MYOVB f | AGTGGGGATGAGGGTTTCAT |
| MYOVB r | TTCCAGCATGGCAGAAACTA |
| CCL2 f | TGTTGTTCACAGTTGCTGCC |
| CCL2 r | GTACTTCTGGACCCATTCCT |
| S100A4 f | GGGGAGAAGGACAGACGAA |
| S100A4 r | GCAGGACAGGAAGACACAG |
| SCNN1G f | CAGCAATGTCCACTTCCATAGG |
| SCNN1G r | CGTTGTAGATGTTTGTGACGGG |
| PADI1 f | ATCCTGGGTCCTGACTTTGG |
| PADI1 r | ACCGATGAGGATTCTGCCGA |

**ADDITIONAL FILE 16:** Oligonucleotides used for experimental validation of expression arrays.
